# Preeclampsia Associated Differences in the Placenta, Fetal Brain, and Maternal Heart Can Be Demonstrated Antenatally: An Observational Cohort Study Using MRI

**DOI:** 10.1161/HYPERTENSIONAHA.123.22442

**Published:** 2024-02-05

**Authors:** Megan Hall, Antonio de Marvao, Ronny Schweitzer, Daniel Cromb, Kathleen Colford, Priya Jandu, Declan P O’Regan, Alison Ho, Anthony Price, Lucy C. Chappell, Mary A. Rutherford, Lisa Story, Pablo Lamata, Jana Hutter

**Affiliations:** 1 Department of Women and Children’s Health (M.H., A.d.M., A.H., L.C.C., L.S.), King’s College London, United Kingdom.; 2 Centre for the Developing Brain (M.H., D.C., K.C., A.H., A.P., M.A.R., L.S., J.H.), King’s College London, United Kingdom.; 3 School of Cardiovascular Medicine (A.d.M., R.S.), King’s College London, United Kingdom.; 4 GKT School of Medical Education (P.J.), King’s College London, United Kingdom.; 5 Centre for Medical Engineering (A.P., P.L.), King’s College London, United Kingdom.; 6 MRC London Institute of Medical Sciences, Imperial College London, United Kingdom (A.d.M., R.S., D.P.O.).; 7 Smart Imaging Lab, Radiological Institute, University Hospital Erlangen, Germany (J.H.).

**Keywords:** hypertension, ischemic heart disease, magnetic resonance imaging, placenta, pregnancy

## Abstract

**BACKGROUND:**

Preeclampsia is a multiorgan disease of pregnancy that has short- and long-term implications for the woman and fetus, whose immediate impact is poorly understood. We present a novel multiorgan approach to magnetic resonance imaging (MRI) investigation of preeclampsia, with the acquisition of maternal cardiac, placental, and fetal brain anatomic and functional imaging.

**METHODS:**

An observational study was performed recruiting 3 groups of pregnant women: those with preeclampsia, chronic hypertension, or no medical complications. All women underwent a cardiac MRI, and pregnant women underwent a placental-fetal MRI. Cardiac analysis for structural, morphological, and flow data were undertaken; placenta and fetal brain volumetric and T2* (which describes relative tissue oxygenation) data were obtained. All results were corrected for gestational age. A nonpregnant cohort was identified for inclusion in the statistical shape analysis.

**RESULTS:**

Seventy-eight MRIs were obtained during pregnancy. Cardiac MRI analysis demonstrated higher left ventricular mass in preeclampsia with 3-dimensional modeling revealing additional specific characteristics of eccentricity and outflow track remodeling. Pregnancies affected by preeclampsia demonstrated lower placental and fetal brain T2*. Within the preeclampsia group, 23% placental T2* results were consistent with controls, these were the only cases with normal placental histopathology. Fetal brain T2* results were consistent with normal controls in 31% of cases.

**CONCLUSIONS:**

We present the first holistic assessment of the immediate implications of preeclampsia on maternal heart, placenta, and fetal brain. As well as having potential clinical implications for the risk stratification and management of women with preeclampsia, this gives an insight into the disease mechanism.

**NOVELTY AND RELEVANCE**

**What Is New?**

We propose a comprehensive magnetic resonance imaging protocol for maternal cardiac and fetal-placental imaging that is safe in pregnancy and acceptable to women, and report on the largest set of functional placental magnetic resonance imaging data in preeclamptic pregnancies.

**What Is Relevant?**

The findings suggest altered oxygenation and microstructure in the placenta, which is associated with similar changes in the fetal brain, and identifies a specific pattern of remodeling in the maternal left ventricle that is associated with preeclampsia.

**Clinical/Pathophysiological Implications?**

As well as giving insight into the immediate pathophysiological impact of preeclampsia, magnetic resonance imaging holds potential as a valuable tool for risk stratification both antenatally, and when considering longer term cardiovascular risk in women.

**See Editorial, pp 848–850**

Preeclampsia occurs when placental malperfusion, and a resultant release of soluble factors into the circulation, causes maternal vascular endothelial injury with subsequent hypertension and multiorgan damage.^1^ Postnatally placental histopathologic correlates with preeclampsia include both villous and vascular lesions,^2^ but sufficient in vivo and in vitro modeling of placental development in disease is lacking, meaning diagnosis is based on maternal symptoms and clinical findings. Incidence is between 2% and 8% with geographic variation.^3^ Globally, 14% of maternal deaths are associated with hypertensive diseases of pregnancy,^4^ which can occur secondary to multiple complications including renal and hepatic failure, stroke, eclampsia, pulmonary edema, and disseminated intravascular coagulopathy. Cardiac manifestations include reduced or increased cardiac output in early- and late-onset disease, respectively,^5^ increased vascular resistance, left ventricular (LV) hypertrophy,^6^ and diastolic dysfunction;^7^ although factors such as whether the disease is early or late onset can impact the severity of associated complications.^8^ After the postnatal period, women who have had preeclampsia remain at an increased risk of vascular disease and ischemic heart disease.^9,10^ Although it is well recognized that maternal biochemical abnormalities can be detected before the clinical onset of preeclampsia,^11^ there is also some evidence that cardiovascular maladaptation precedes the diagnosis of preeclampsia: echocardiography has demonstrated abnormalities in LV mass in the late third trimester before the development of term preeclampsia.^12,13^

For the fetus, preeclampsia is associated with fetal growth restriction and complications such as placental abruption; excluding congenital anomalies, 5% of stillbirths occur in women with preeclampsia. In infants born to women with preeclampsia, increased rates of hypertension and cardiac dysfunction, stroke, cognitive dysfunction, and psychiatric morbidity have been demonstrated.^14–16^

Cardiac magnetic resonance imaging (MRI) combines excellent spatial and temporal resolution and is the gold-standard noninvasive method of assessing cardiac morphology and function. In contrast with echocardiography, cardiac MRI is not limited by geometric assumptions in assessing ventricular volumes and image acquisition is less operator dependent. This allows highly reproducible and accurate assessment of cardiac structure and function,^17^ making it the ideal modality for assessing differences between small groups.^18^ We have provided pilot data demonstrating the feasibility of cardiac MRI in pregnancy, but large prospective studies are still required.^19^

MRI also offers an opportunity for in vivo study of the placenta in preeclampsia, as well as evaluation of maternal and fetal structures. Recent studies applying functional MRI techniques to the placenta have proposed reduced oxygenation in the preeclamptic placenta via demonstration of increased heterogeneity and decreased mean T2*. T2* is an MRI technique that discriminates between oxygenated and deoxygenated hemoglobin, so allowing a proxy assessment of relative oxygenation with a lower value indicating a higher level of deoxygenated hemoglobin.^20,21^ There is evidence of decreased placental diffusivity in small for gestational age fetuses, suggesting less free movement of water molecules.^22,23^ Furthermore, advances in acquisition and reconstruction techniques have allowed more detailed investigation of the fetal brain.^24^

We aim to provide a multiorgan approach to MRI investigation of preeclampsia with the acquisition of maternal cardiac, placental, and fetal brain anatomic and functional imaging. We hypothesize that in pregnancies affected by preeclapmsia, there will be a reduction in placental T2* with an associated reduction in fetal brain T2*. We further hypothesize that evidence of the chronic LV remodeling noted to occur in women who have had preeclampsia may in fact be evident antenatally. We include group of pregnant women with chronic hypertension to discriminate further between physiological changes, hypertensive changes, and those specifically relating to preeclampsia. To improve construct validity, we include a cohort of nonpregnant women in our statistical shape analysis.

## METHODS

A prospective observational study was performed between 2019 and 2022 at Guy’s and St Thomas’ National Health Service Foundation Trust (London Dulwich Ethics Committee 08/LO/1958). Additional high-risk placental and fetal data sets were obtained from a previous study (London Dulwich Ethics Committee 16/LO/1573). Additional nonpregnant control data sets were obtained from a previous study, the UK Digital Heart Project (London – West London & Gene Therapy Advisory Committee Research Ethics Committee 17/LO/0034). Written, informed consent was obtained from all participants.

Women were recruited from antenatal clinics into 3 categories: pregnant controls, women with preeclampsia, and pregnant women with chronic hypertension; women were retrospectively excluded if they had been recruited as controls but had developed other pregnancy complications. The chronic hypertensive group was included to insure that observed differences were due to preeclampsia as opposed to any hypertensive disease in pregnancy. Women with diabetes and a hypertensive disorder were analyzed within their hypertensive cohort, although any women who had significant maternal or fetal complications associated with diabetes or who had rapidly increasing pharmacological requirements were not included. For all groups inclusion criteria included gestational age 18 to 42 weeks, a body mass index<40 kg/m^2^ (with the MRI bore size the limiting factor), age 16 to 50 years, and no contraindication to MRI such as noncompatible metal implants or claustrophobia. All women were offered up to 3 scans so as to assess longitudinal change, although only had to undergo one to be included in the analysis.

Case inclusion criteria were a diagnosis of preeclampsia (including superimposed on chronic hypertension) defined by the International Society for the Study of Hypertension in Pregnancy as hypertension with one of the following new-onset conditions at 20 weeks’ gestation or beyond: proteinuria, acute kidney injury, hepatic dysfunction, neurological features, hemolysis or thrombocytopenia, or fetal growth restriction.^25^ Women with chronic hypertension were recruited as a separate cohort.

Demographic and clinical information, including on previous obstetric history, was collected on all participants. For women with preeclampsia and chronic hypertension, the results of nearest routine fetal growth and Dopplers to the MRI were recorded; in particular, this allows for comparison of fetal brain MRI findings to Doppler-based evidence of redistribution (a well-recognized phenomenon whereby fetal hemodynamics alter to redistribute blood flow towards the brain when in a relatively hypoxic environment. Middle cerebral artery pulsatility index below the 5th centile or a cerebroplacental ratio below the 5th centile were used to define cerebral redistribution; while the middle cerebral artery was used clinically to define redistribution in these patients, the decision to include the cerebroplacental ratio is reflective of its higher specificity in detecting fetal hypoxia).^26,27^ Fetal growth restriction was defined as per the 2016 Delphi Consensus on the definition of fetal growth restriction.^26^ Neonatal outcomes including gestation at birth, mode of delivery, condition at birth, need for neonatal support, and birthweight were collected. Placental histopathology was collected; lesions associated with preeclampsia were noted as per the Amsterdam Criteria (maternal vascular malperfusion lesions of the placental bed, including infarcts, retroplacental hemorrhage, distal villous hypoplasia, accelerated villous maturation, and decidual arteriopathy).^28^As this was a pilot study relying on involvement of women with a complex and acute medical condition in pregnancy, there is no predefined sample size.

A cohort of nonpregnant women was analyzed as part of the statistical shape model to add construct validity and avoid over fitting. This group included women with no cardiovascular disease or risk factors. Propensity score matching was undertaken in R MatchIt, although 1:1 matching was not possible owing to underrepresentation of women of African ancestry in the nonpregnant cohort.

### Magnetic Resonance Imaging

After informed consent was obtained, participants had an MRI on a clinical 1.5T Philips Ingenia scanner using a combined 24-channel posterior and torso (dStream) coil. Maternal comfort in the supine position was achieved by careful positioning of the head and legs on elevated cushions, back padding, and additional cushions as requested. The MRI was performed in 2 sessions (maternal cardiac and fetal placental) each of which lasted around 30 minutes. A break was given in the middle of the session. To reduce any bias that could arise from maternal anxiety at the start of the scan (eg, tachycardia or hypertension), women with an odd case study ID underwent fetal-placental imaging first, and those with an even ID underwent cardiac imaging first.

An obstetrician or midwife was present for all scans. Maternal temperature was recorded before and after the scan. Continuous oxygen saturations were measured, and blood pressure was taken every 10 minutes.

The protocol is illustrated in Figure S1 and details of all individual sequences provided in Supplemental text and Table S1; an example of images acquired is given in Figure S2.

Nonpregnant controls from the healthy control group underwent an equivalent cardiac MRI protocol on a 1.5-T Philips Achieva system (Best, the Netherlands) as previously described.^29^

### Analysis

All analyses were undertaken by operators masked to the clinical diagnosis. The placenta was visually inspected on a T2 image and reported in line with our previous work.^30^ Manual outlining was performed on a functional scan mapped to subsequent scans by J. Hutter (interclass correlation with A. Ho previously confirmed as 0.92). Monoexponential fitting was performed to obtain T2* maps. Mean placenta T2*, histogram skewness, and kurtosis were calculated.

Brain volume (supratentorial tissues excluding brainstem and cerebrospinal fluid) was manually segmented on functional maps by D. Cromb (interclass correlation previously confirmed as 0.811). Mean T2* values were obtained by averaging the T2* maps from manually outlined brains.

The cardiac MRIs were analyzed using cvi42 postprocessing software (Version 5.1.4; Circle Cardiovascular Imaging, Inc, Calgary, Canada), using standard clinical methodology.^31^

The three-dimensional (3D) LV morphology was studied by the construction of a statistical shape model from the segmentations of the short axis stack at the end-diastolic frame as reported in previous studies.^32–34^ Briefly, 3D meshes were built using a computational anatomy tool kit,^35^ anatomic modes of variation were found by principal component analysis, and the impact of preeclampsia was assessed by an optimized linear discriminant analysis of the first 12 modes.

Flow data were processed using CVI42 Flow version 5.10.3. The magnitude image with the sharpest contrast was used to determine vessel contours. Contours were then propagated to phase contrast images (with manual correction as required) in all temporal phases.

All parameters were first assessed against gestational age, and then as cases against controls. Matching was undertaken to nearest gestational age, with further analysis using the Mann-Whitney *U* test.

### Statistical Analysis

Statistical analysis was performed with R version 3.6.0 (R Foundation for Statistical Computing) and RStudio Server version 1.043. Normality was confirmed on histograms. Variables are expressed as percentages if categorical, mean±SD if continuous and normal, and median (interquartile range) if continuous and nonnormal. Baseline anthropometric data were compared by using Kruskal-Wallis tests and, if differences were identified, a Wilcoxon test was used for pairwise comparisons with Benjamini-Hochberg adjustment for multiple testing. Imaging parameters in ≥2 groups were compared by using ANCOVA, adjusted for relevant clinical covariates including gestational age at time of scan. When differences were significant, a Tukey post hoc test was applied for pairwise multiple comparisons.

## RESULTS

### Study Participants

Seventy-eight scans were obtained from 65 pregnant women (with 9 women having 2 scans, and 2 having 3 scans). An additional 38 scans were obtained from healthy nonpregnant controls (demographic characteristics provided in Table S2). Cardiac assessment was not completed in 2 pregnant participants: one owing to claustrophobia, and one to worsening hypertension. For a further 16 women, no cardiac data were obtained. Nine women developed pregnancy complications after being scanned as a control and so were excluded. Thirteen women with preeclampsia were recruited, 1 of whom had 3 MRIs. Of the 13 women, 6 had preeclampsia superimposed on chronic hypertension. Table 1 summarizes the demographics of all cohorts. Table S3 details the relevant obstetric history and outcomes of the preeclamptic cohort.

**Table 1.**
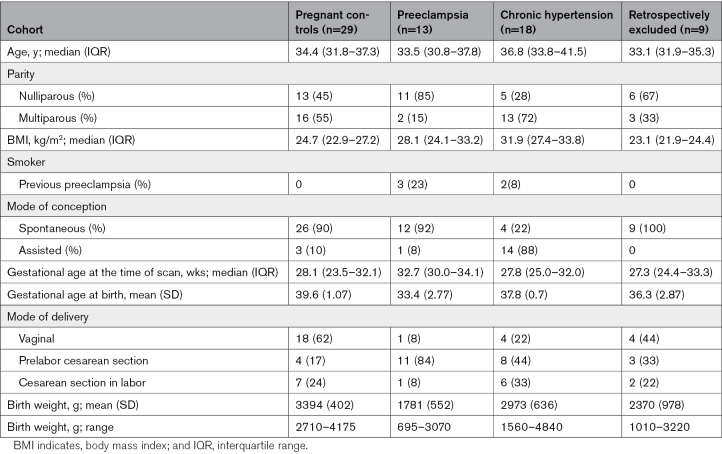
Demographics of Participants

### Placenta

**Figure 1.**
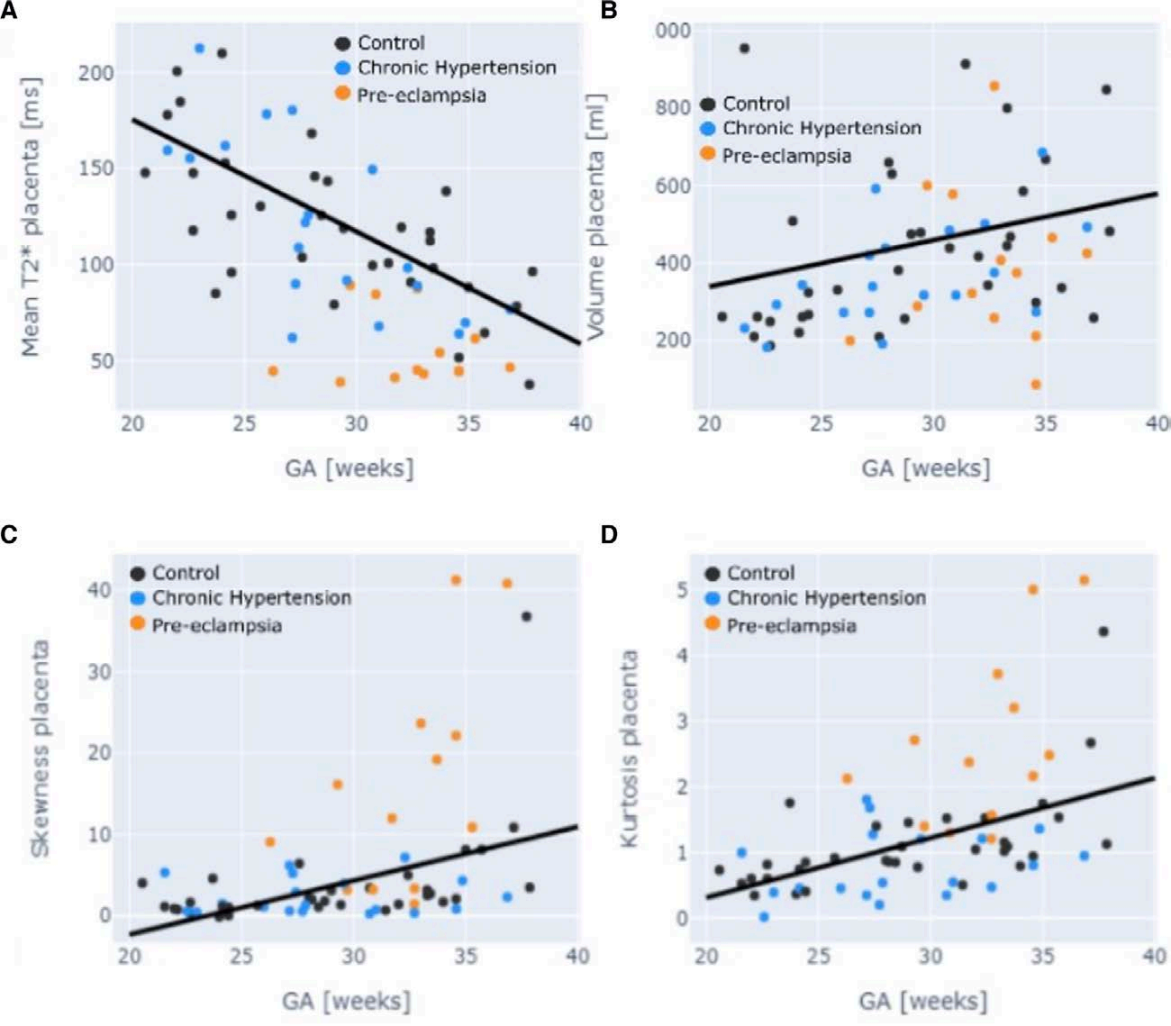
Placental anatomic and T2* by group. A, Mean placental T2* by gestation. **B**, Placental volume by gestation. **C**, Skewness. **D**, Kurtosis by gestation. Black dot denotes control; blue denotes chronic hypertension; and orange denotes preeclampsia. GA indicates gestational age.

In the crosssectional control group, there was a decrease in mean T2* values across gestation (*P*<0.01). The decrease in mean T2* in the chronic hypertensive group did not reach significance. The preeclamptic cohort had significantly lower T2* values throughout gestation, and a gradual decline was not seen. Z scores for all 3 groups were calculated for both mean placental T2* and placental volume (Figure 1A and 1B). For mean T2*, the Z scores obtained were controls=0.00+–1.03; chronic hypertension, –0.71+–0.7 (P=0.2); preeclampsia, –1.88+–0.75 (*P*<0.001); for the placental volume for controls 0.00+–1.03; for chronic hypertension, –0.31+–1.025 (P=0.4); for the preeclampsia, –0.54+–1.08 (*P*=0.05).

Both skewness and kurtosis increased throughout gestation in the control group, with the chronic hypertensive group overlying this, although not reaching significance. The preeclamptic group had higher skewness and kurtosis than both other groups, and placental volume was reduced (*P*=0.02; Figure 1). The Z scores for skewness for the chronic hypertension group were –1.46+–3.06 and for preeclampsia, 1.64+–1.75 for kurtosis in the chronic hypertension cohort, –0.26–0.77 and for preeclampsia, 1.67+–1.45 (Figure 1C and 1D).

Twenty-three percent with preeclampsia had placental T2* values with a Z score of ≥–1; of note these were the only women without any preeclampsia-related abnormalities on placental histopathology (Table 2; Table S3). These findings were unchanged by the removal of longitudinal datasets from the analysis.

**Table 2.**
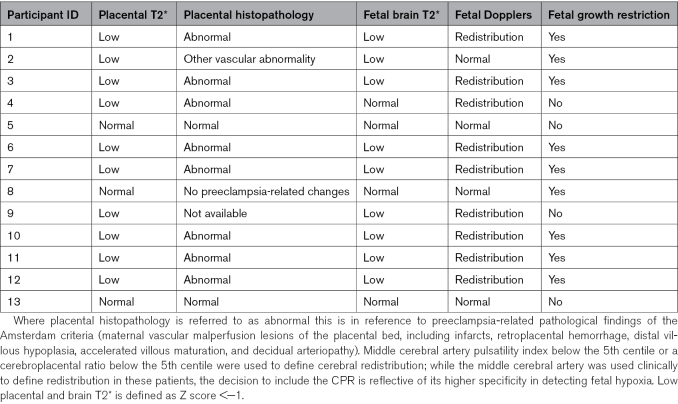
Relationship Between Fetal Brain and Placental T2* and Clinical Parameters in Patients With Preeclampsia

Visual inspection of control versus preeclamptic placentas revealed a more variable lobule size and less consistent signal intensity in women with preeclampsia. There was also an increase in low signal areas as compared with controls.

### Fetal Brain

**Figure 2.**
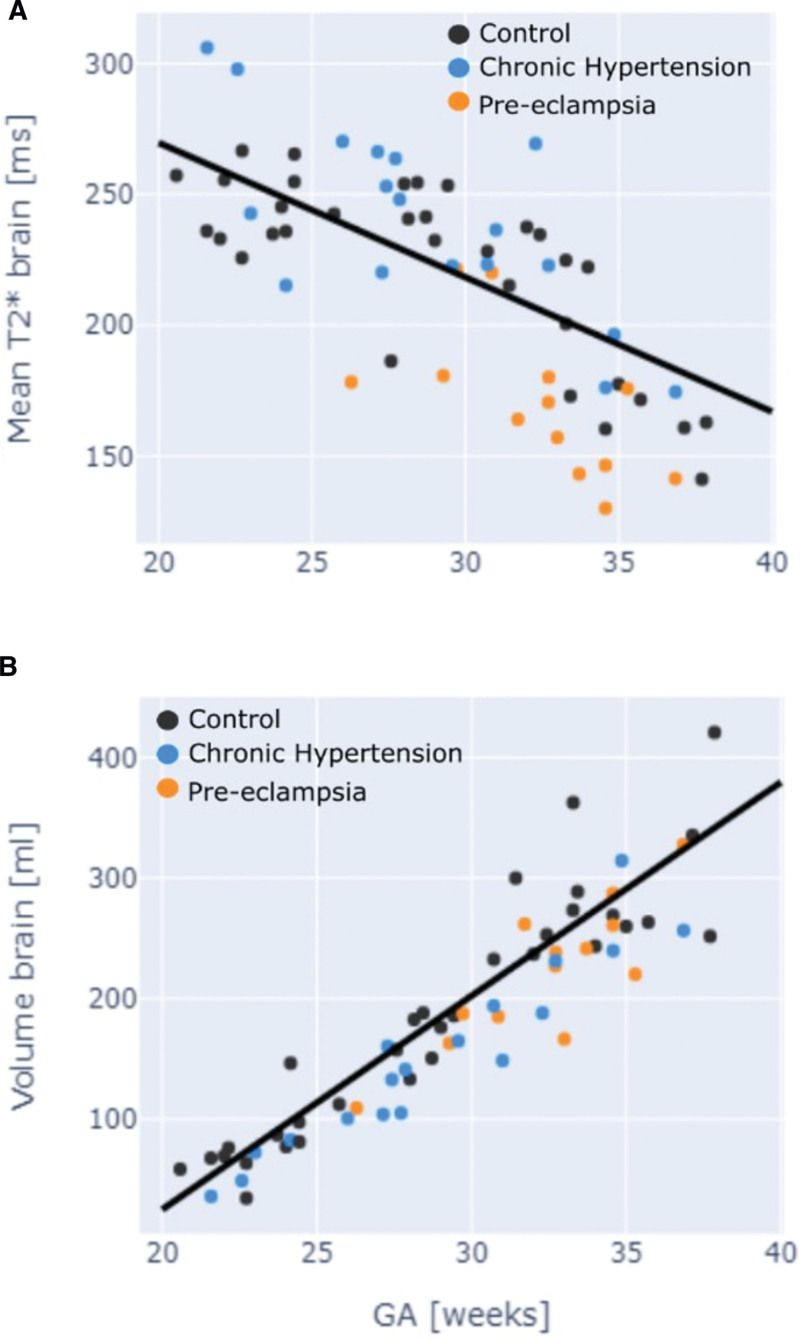
Fetal brain anatomic and T2* by group. A, Fetal brain mean T2* by gestation. **B**, Fetal brain volume by gestation. Black dot denotes control; blue denotes chronic hypertension; orange denotes preeclampsia.

**Figure 3.**
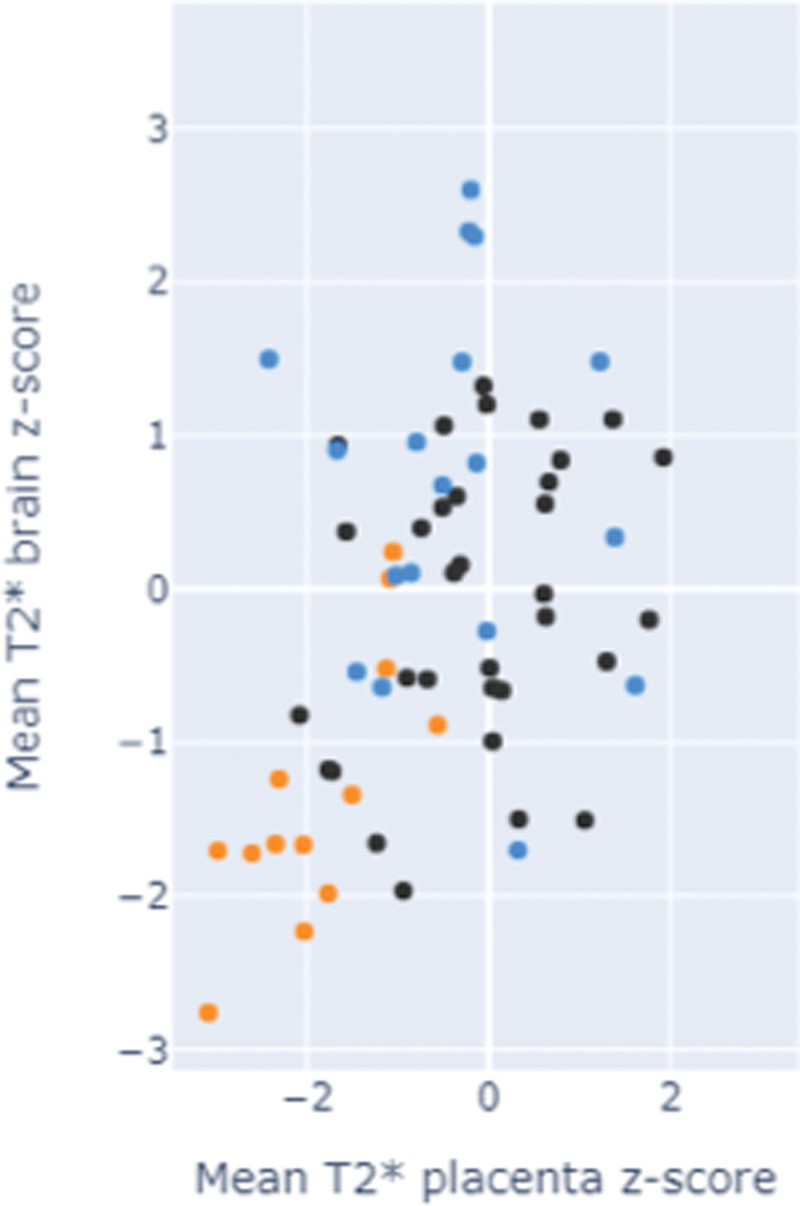
Correlation of placental and brain T2* Z-scores. Black dots denotes control; blue denotes chronic hypertension; and orange denotes preeclampsia.

Fetal brain T2* values were demonstrated to decline with advancing gestation among controls. This relationship was preserved in the chronic hypertensive group. Among women with preeclampsia, the fetal brain T2* values were significantly reduced through all gestations (*P*<0.01) and did not have a linear decline with gestation (Figure 2). Z scores for all 3 groups were as follows: control, 0.0+−0.91; chronic hypertension, −0.65+−0.94 (*P*=0.4); and preeclampsia, −1.34+−0.84 (*P*<0.001; Figure 3) Among preeclamptic participants, 31% of cases had brain T2* was in line with controls; in 75% these cases fetal Doppler studies were normal (Table 2; Table S3). One case demonstrated an abnormal T2* values but with preserved fetal Doppler studies (Table 2; Table S3). Brain volume was reduced in the presence of preeclampsia (*P*=0.03; Figure 2). These findings were unchanged by removal of longitudinal data sets from the analysis.

Brain and placental T2* Z scores showed a direct correlation between normal and low scores in both domains (Figure 3).

Major differences in the placental and fetal findings of the control and preeclampsia groups are summarized in Figure S3.

### Cardiac MR

Systolic (*P*<0.001) and diastolic (*P*=0.03) blood pressure were lower in the pregnant controls (107/66 mm Hg) than in preeclamptic participants (121/77 mm Hg). There were no other baseline anthropometric differences between groups.

**Figure 4.**
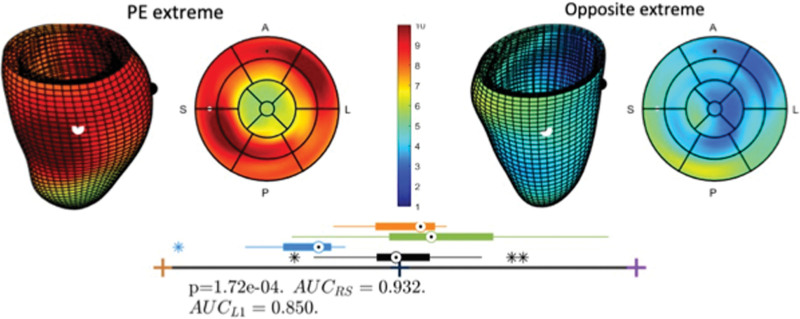
**Left ventricular remodeling associated with preeclampsia.** Left ventricular remodeling associated with preeclampsia (PE), illustrating thickened walls and eccentricity and bulging located at the outflow track. **Top**, Extremes of the anatomic mode that best discriminate pregnant controls to pregnant preeclamptic subjects. The 3-dimensional and bullseye views are color-coded by thickness (in mm) and have black and white spheres at the sides of the outflow track for easy mapping between both views. **Bottom**, Box-plot of distributions of pregnant controls (black, n=26), preeclampsia (blue, n=9), chronic hypertension (orange, n=4) and nonpregnant controls (green), together with metrics of the discriminant performance of the linear discriminant analysis between pregnant controls and preeclampsia. A indicates anterior; AUC, area under the curve; L, lateral; L1, leave-1 out cross-validation; P, posterior; RS, resubstitution; and S, septal.

The LV mass of the preeclamptic group (98.05±24.5 g) was higher than both the pregnant controls (83.3±12.08 g; *P*=0.04) but not different from those with chronic hypertension (89.3±18.8; *P*=0.54)). The study of the 3D anatomy revealed a specific thickening pattern (existence of regional increase in wall thickness wall locations of mid anterolateral and posteroseptal) together with changes in eccentricity (the ventricular cross-section displayed a dilation in the axis oriented in the direction of the outflow tract) and the onset of a bulge below the outflow track, see Figure 4. A detailed inspection of the modes of anatomic variation further reveals that the thickening pattern associated to preeclampsia was linked to a localized basal concentric remodeling and not to the complementary basal eccentric remodeling (see modes 11 and 13 in Figure S4).

## DISCUSSION

### Summary of Main Findings

We have demonstrated a comprehensive anatomic and functional MRI protocol for cardiac and fetal-placental imaging that is safe in the second and third trimester of pregnancy and acceptable to women, with 97% of scans completed. As far as we are aware, this is the largest set of functional placental MRI data in preeclamptic pregnancies, and confirms previous findings suggestive of altered oxygenation and microstructure. We have demonstrated altered T2* in the fetal brain, in proportion to that seen in the placenta. Finally, we have characterized the remodeling pattern of the maternal left ventricle associated with the presence of preeclampsia. Conventional cardiac MRI analysis demonstrated higher LV mass in preeclampsia than in uncomplicated pregnancies, while 3D statistical modeling revealed specific characteristics of eccentricity and outflow track remodeling beyond the thickening of the walls.

### Comparison to Other Work

The placental phenotype in control pregnancies is in line with previous work: the reduction in T2* across gestation in normal pregnancies has been demonstrated using both our imaging protocols^36^ and those of others.^37,38^ As previously demonstrated, there is overlap between chronic hypertensive pregnancies and normal pregnancies in terms of mean T2*, skewness, and kurtosis.^20^ Pregnancies affected by preeclampsia are typically outliers in all domains. This is to be expected given the higher rate of hypoxic vascular anomalies seen in the preeclamptic placenta compared with that affected by chronic hypertension alone.^39^

In all cases where the placental T2* in preeclamptic pregnancies lay within the normal range, there was normal placental histopathology and delivery at later gestations, suggesting a less severe clinical phenotype. Preexisting work on placental perfusion MRI in women with early and late preeclampsia, has demonstrated preserved placental perfusion in women with later onset disease.^40^ The decreased placental perfusion seen in the early preeclampsia group, as well as histopathologic evidence for greater maternal malperfusion in early-onset preeclampsia as compared with late onset,^41^ provide basis for the variation seen in our cohort. Whether this is due only to differences in the placenta, or whether the cause of late-onset preeclampsia is perhaps more driven by maternal cardiovascular changes rather than placental pathology remains uncertain.^7^ Nonetheless, our findings go further to support the ability of functional placental MRI to differentiate between disease severities.

Novel to this study is the association between placental T2* and fetal brain T2*. The negative correlation between decreasing brain T2* and increasing gestation in the normal pregnancy is already established and thought to be secondary to many structural changes including decreasing water density and increasing myelination.^24^ Preservation of brain volume with a decrease in T2* in the preeclampsia group may reflect a reduction in oxygenation, with or without reduction in angiogenesis. This seems particularly likely given that this does not seem to occur in the absence of reduced placental T2*, and that in all but 1 case there is corresponding Doppler evidence of cerebral redistribution. Long-term neurodevelopmental and behavioral outcomes of children born to mothers with preeclampsia are recognized, and a small study has shown differences in neuroanatomy persisting at 7 to 10 years;^42^ although this is difficult to disentangle from the effects of prematurity, our findings may suggest an antenatal antecedent.

Looking into the mother’s health, novel to this study is the 3D thickening pattern of the left ventricle that is found discriminant of the presence of preeclampsia. The extra workload in preeclampsia is indeed increasing the cardiac mass and mass-to-volume ratio, as previously reported in much larger echocardiography studies.^6^ This demonstrates the higher power of cardiac MRI to identify differences between small groups, and its role in cardiovascular research in pregnancy. Our preliminary data further reveal an intriguing pattern of thickening, eccentricity and bulging as illustrated in Figure 4. The fact that the chronic hypertension group did not show differences with control pregnancy along this axis suggests that this is an acute maladaptive remodeling process specific to preeclampsia. In relationship with this finding, the chronic remodeling of the left ventricle 5 to 10 years after a hypertensive pregnancy manifests as a global concentric (and not eccentric) thickening of the left ventricle.^43^ Intriguingly in the response of preeclampsia during pregnancy, it is only the base of the heart where this concentric (and not eccentric) thickening pattern is seen (see Figure S1). This collection of observations leads us to hypothesize that it is the base of the heart the region that is first recruited and most intensively working in response to preeclampsia, and that the rapid onset of hypertension causes a level of uneven thickening pattern and septal bulging. Given that this study includes both early- and late-onset cases of preeclampsia the similarity in cardiac output between preeclampsia and control groups is to be expected.

### Strengths and Limitations

This study is the largest to date using MRI to investigate maternal, fetal, and placental impactions of preeclampsia, using an optimized protocol that is safe and acceptable to women. The data are made more robust by consistent positioning and imaging protocols, as well as postprocessing including motion correction. The inclusion of women with chronic hypertension demonstrates where phenotypes are driven by preeclampsia specifically.

Limitations include the relatively small number of women with preeclampsia included, reflecting the difficulty recruiting given the often short interval from diagnosis to delivery; this may obscure significant differences between groups, but also limits any comments that can be made on confounding factors in cases such as diabetes and timing of disease onset. Given the small numbers of women with preeclampsia included and the variation in phenotype displayed, a multidimensional analysis of the heart, placenta, and fetal brain is not plausible at this stage.

### Implications

Although clinical fetal MRI is largely confined to the assessment of structural anomalies, there is evidence here of a potential role for other high-risk obstetric conditions. We have demonstrated feasibility of a comprehensive fetal, placental, and maternal cardiac MRI in terms of safety, data acquisition, and acceptability to women.

Future work into determining risk of developing, likely clinical severity and implications of preeclampsia should combine MRI, obstetric ultrasound, and biomarkers, such as placental growth factor, as it is likely that a combination of investigations could yield greatest information regarding initial diagnosis as well as predicted course of disease.

Although we have demonstrated a correlation of findings associated with preeclampsia, further work should be performed in defining this phenotype. As well as increasing the cohort size, attention should be paid to early- and late-onset disease, as these are likely to be mechanistically different and have different clinical end point.^44^ Furthermore, an increase in cohort size would allow for separate analysis of previously normotensive women who develop preeclampsia, and women with preexisting chronic hypertension who develop superimposed preeclampsia. Although fetal growth restriction has previously been associated with reduced placental T2*,^23,45^ we have been unable to delineate the implications of preeclampsia on this finding. Creation of a cohort where the interplay between these 3 findings could be investigated would be of value. MRI could have implications for earlier diagnosis of preeclampsia; high-risk women (eg, those with early-onset growth restriction) who are not yet diagnosed with preeclampsia should be included in future work, as this could have significant implications for their management. Moreover, the 3D-specific remodeling pattern linked to preeclampsia could have implications for prognosis and risk models for future cardiovascular events. Any future work on cardiac imaging should include longitudinal and postnatal data to better delineate the mechanisms of continued cardiac risk, and for stratification of risk in individuals.

In terms of perinatal outcomes, we have demonstrated a relationship between preeclampsia and reduced fetal brain T2*, and also between reduced fetal brain T2* and fetal cerebral Doppler redistribution. Although there is some evidence of abnormal neurocognitive outcomes in children who have been affected by redistribution,^46^ further neurocognitive follow-up of children with paired MRI and ultrasound data could improve understanding of the longer term clinical implications of preeclampsia on the child.

In terms of techniques used, while the relationship between T2* and deoxyhemoglobin is well established, it is subject to influence from other factors. Therefore, additional functional imaging, such as diffusion techniques, linked with recent advanced analysis techniques^47^ may lead to greater insight of the mechanisms of preeclampsia.

### Perspectives

We demonstrate that MRI may provide maternal and fetal insights into preeclampsia that cannot be obtained by other means during pregnancy; and that these can be obtained in a clinical MRI scanner in an examination that is acceptable to women. Functional placental MRI may be able to differentiate between preeclampsia disease severities, whereas fetal brain imaging may help refine neurodevelopmental prognostic assessment of children exposed to preeclampsia in utero, as well as give insight into pathological pathways underlying the changes seen in this group. In our small sample, specific 3D pattern of LV remodeling and thickening was found to be discriminant of the presence of preeclampsia and may provide a basis for improved clinical risk stratification. Confirmation of these findings in a larger group of women is required before inclusion in risk-stratification models.

## ARTICLE INFORMATION

### Acknowledgments

The authors are grateful to the women who participated in this study. This work is the opinion of the authors and not necessarily that of the National Health Service, National Institute for Health Research, or Department of Health.

### Sources of Funding

This work was supported by core funding from the Wellcome/Engineering and Physical Sciences Research Council Center for Medical Engineering (WT203148/Z/16/Z), by the National Institutes of Health Human Placenta Project grant 1U01HD087202-01 (Placenta Imaging Project), by the Wellcome Trust, Sir Henry Wellcome Fellowship to J. Hutter (201374/Z/16/Z), Senior Research Fellowship to P. Lamata (209450/Z/17/Z), National Institute for Health Research Imperial College Biomedical Research Center, Medical Research Council (MC_UP_1605/13), British Heart Foundation (RG/19/6/34387, RE/18/4/34215), Academy of Medical Sciences (SGL015/1006) and Fetal Medicine Foundation (495237).

### Disclosures

None.

## Supplementary Material
