# Pre-eclampsia associated differences in the placenta, fetal brain and maternal heart can be demonstrated antenatally: An observational cohort study using MRI

**DOI:** 10.1161/HYPERTENSIONAHA.123.22442

**Published:** 2024-02-05

**Authors:** Megan Hall, Antonio de Marvao, Ronny Schweitzer, Daniel Cromb, Kathleen Colford, Priya Jandu, Declan P O’Regan, Alison Ho, Anthony Price, Lucy C. Chappell, Mary A. Rutherford, Lisa Story, Pablo Lamata, Jana Hutter

**Affiliations:** 1 Department of Women and Children’s Health, King’s College London, UK; 2 Centre for the Developing Brain, King’s College London, UK; 3 School of Cardiovascular Medicine, King’s College London, UK; 4 MRC London Institute of Medical Sciences, Imperial College London, UK; 5 GKT School of Medical Education, King’s College London, UK; 6 Centre for Medical Engineering, King’s College London, UK; 7 Smart Imaging Lab, Radiological Institute, University Hospital Erlangen, Erlangen, Germany

**Keywords:** Pre-eclampsia, hypertension in pregnancy, fetal MRI, cardiac MRI, functional MRI, placental MRI

## Abstract

**Background:**

Pre-eclampsia is a multiorgan disease of pregnancy that has short- and long-term implications for the woman and fetus, whose immediate impact is poorly understood. We present a novel multi-organ approach to MRI investigation of pre-eclampsia, with acquisition of maternal cardiac, placental, and fetal brain anatomical and functional imaging.

**Methods:**

An observational study was carried out recruiting three groups of pregnant women: those with pre-eclampsia, chronic hypertension, or no medical complications. All women underwent a cardiac MRI, and pregnant women underwent a placental-fetal MRI. Cardiac analysis for structural, morphological and flow data was undertaken; placenta and fetal brain volumetric and T2* (which describes relative tissue oxygenation) data were obtained. All results were corrected for gestational age. A non-pregnant cohort was identified for inclusion in the statistical shape analysis.

**Results:**

Seventy-eight MRIs were obtained during pregnancy. Cardiac MRI analysis demonstrated higher left ventricular mass in pre-eclampisa with 3D modeling revealing additional specific characteristics of eccentricity and outflow track remodelling. Pregnancies affected by pre-eclampsia demonstrated lower placental and fetal brain T2*. Within the pre-eclampsia group, 23% placental T2* results were consistent with controls, these were the only cases with normal placental histopathology. Fetal brain T2* results were consistent with normal controls in 31% of cases.

**Conclusions:**

We present the first holistic assessment of the immediate implications of pre-eclampsia on maternal heart, placenta and fetal brain. As well as having potential clinical implications for the risk-stratification and management of women with pre-eclampsia, this gives an insight into disease mechanism.

## Graphical abstract

**Graphical Abstract:**
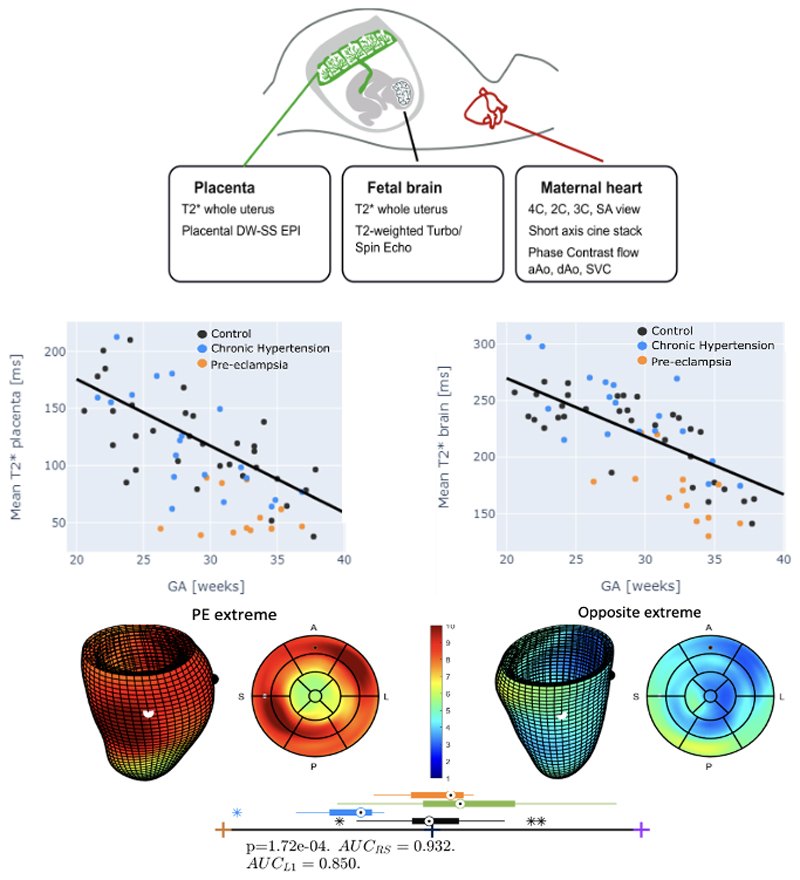

## Introduction

Pre-eclampsia occurs when placental malperfusion, and a resultant release of soluble factors into the circulation, causes maternal vascular endothelial injury with subsequent hypertension and multi-organ damage.^1^ Postnatally placental histopathological correlates with pre-eclampsia include both villous and vascular lesions,^2^ but sufficient *in vivo* and *in vitro* modelling of placental development in disease is lacking, meaning diagnosis is based on maternal symptoms and clinical findings. Incidence is between 2 and 8%, with geographical variation.^3^ Globally, 14% of maternal deaths are associated with hypertensive diseases of pregnancy,^4^ which can occur secondary to multiple complications including renal and hepatic failure, stroke, eclampsia, pulmonary oedema, and disseminated intravascular coagulopathy. Cardiac manifestations include reduced or increased cardiac output in early and late onset disease respectively,^5^ increased vascular resistance, left ventricular hypertrophy,^6^ and diastolic dysfunction;^7^ although factors such as whether the disease is early or late onset can impact the severity of associated complications.^8^ After the postnatal period, women who have had pre-eclampsia remain at an increased risk of vascular disease and ischaemic heart disease.^9,10^ While it is well recognised that maternal biochemical abnormalities can be detected prior to clinical onset of pre-eclampsia,^11^ there is also some evidence that cardiovascular maladaptation precedes the diagnosis of pre-eclampsia: echocardiography has demonstrated abnormalities in left ventricular mass in the late third trimester prior to the development of term pre-eclampsia.^12,13^

For the fetus, pre-eclampsia is associated with fetal growth restriction (FGR) and complications such as placental abruption; excluding congenital anomalies, 5% of stillbirths occur in women with pre-eclampsia In infants born to women with pre-eclampsia, increased rates of hypertension and cardiac dysfunction, stroke, cognitive dysfunction and psychiatric morbidity have been demonstrated.^14–16^

Cardiac magnetic resonance imaging (MRI) combines excellent spatial and temporal resolution, and is the gold-standard non–invasive method of assessing cardiac morphology and function. In contrast with echocardiography, cardiac MRI is not limited by geometric assumptions in assessing ventricular volumes and image acquisition is less operator-dependent. This allows highly reproducible and accurate assessment of cardiac structure and function,^17^ making it the ideal modality for assessing differences between small groups.^18^ We have provided pilot data demonstrating feasibility of cardiac MRI in pregnancy, but large prospective studies are still required.^19^

MRI also offers an opportunity for *in vivo* study of the placenta in pre-eclampsia, as well as evaluation of maternal and fetal structures. Recent studies applying functional MRI techniques to the placenta have proposed reduced oxygenation in the pre-eclamptic placenta via demonstration of increased heterogeneity and decreased mean T2*. T2* is an MRI technique that discriminates between oxygenated and deoxygenated haemoglobin, so allowing a proxy assessment of relative oxygenation with a lower value indicating a higher level of deoxygenated haemoglobin.^20,21^ There is evidence of decreased placental diffusivity in small for gestational age fetuses, suggesting less free movement of water molecules.^22,23^ Furthermore, advances in acquisition and reconstruction techniques have allowed more detailed investigation of the fetal brain.^24^

We aim to provide a multi-organ approach to MRI investigation of pre-eclampsia with acquisition of maternal cardiac, placental, and fetal brain anatomical and functional imaging. We hypothesise that in pregnancies affected by pre-eclapmsia there will be a reduction in placental T2* with an associated reduction in fetal brain T2*. We further hypothesise that evidence of the chronic left ventricular remodelling noted to occur in women who have had pre-eclampsia may in fact be evident antenatally. We include group of pregnant women with chronic hypertension in order to discriminate further between physiological changes, hypertensive changes and those specifically relating to pre-eclampsia. To improve construct validity we include a cohort of non-pregnant women in our statistical shape analysis.

## Methods

A prospective observational study was performed between 2019 and 2022 at Guy’s and St Thomas’ NHS Foundation Trust (London Dulwich Ethics Committee 08/LO/1958). Additional high-risk placental and fetal datasets were obtained from a previous study (London Dulwich Ethics Committee 16/LO/1573). Additional non-pregnant control datasets were obtained from a previous study, the UK Digital Heart Project (London - West London & GTAC Research Ethics Committee 17/LO/0034). Written, informed consent was obtained from all participants.

Women were recruited from antenatal clinics into three categories: pregnant controls, women with pre-eclampsia, and pregnant women with chronic hypertension; women were retrospectively excluded if they had been recruited as controls but had developed other pregnancy complications. The chronic hypertensive group was included to insure that observed differences were due to pre-eclampsia as opposed to any hypertensive disease in pregnancy. Women with diabetes and a hypertensive disorder were analysed within their hypertensive cohort, although any women who had significant maternal or fetal complications associated with diabetes or who had rapidly increasing pharmacological requirements were not included. For all groups inclusion criteria included gestational age 18-42 weeks, a body mass index (BMI) <40 kg/m^2^ (with the MRI bore size the limiting factor), aged 16-50 years, and no contraindication to MRI such as non-compatible metal implants or claustrophobia. All women were offered up to three scans so as to assess longitudinal change, although only had to undergo one in order to be included in analysis.

Case inclusion criteria was a diagnosis of pre-eclampsia (including superimposed on chronic hypertension) defined by the International Society for the Study of Hypertension in Pregnancy as hypertension with one of the following new-onset conditions at 20 weeks’ gestation or beyond: proteinuria, acute kidney injury, hepatic dysfunction, neurological features, haemolysis or thrombocytopaenia, or fetal growth restriction^25^. Women with chronic hypertension were recruited as a separate cohort.

Demographic and clinical information, including on previous obstetric history, was collected on all participants. For women with pre-eclampsia and chronic hypertension the results of nearest routine fetal growth and Dopplers to the MRI were recorded; in particular this allows for comparison of fetal brain MRI findings to Doppler based evidence of redistribution (a well-recognised phenomenon whereby fetal haemodynamics alter in order to redistribute blood flow towards the brain when in a relatively hypoxic environment. Middle cerebral artery pulsatility index below the 5th centile or a cerebroplacental ratio below the 5th centile were used to define cerebral redistribution; while the middle cerebral artery was used clinically to define redistribution in these patients, the decision to include the cerebroplacental ratio is reflective of its higher specificity in detecting fetal hypoxia).^26,27^ Fetal growth restriction was defined as per the 2016 Delphi Consensus on the definition of fetal growth restriction.^26^ Neonatal outcomes including gestation at birth, mode of delivery, condition at birth, need for neonatal support, and birthweight were collected. Placental histopathology was collected; lesions associated with pre-eclampsia were noted as per the Amsterdam Criteria (maternal vascular malperfusion lesions of the placental bed, including infarcts, retroplacental haemorrhage, distal villous hypoplasia, accelerated villous maturation, and decidual arteriopathy*)*.^28^As this was a pilot study relying on involvement of women with a complex and acute medical condition in pregnancy, there is no pre-defined sample size.

A cohort of non-pregnant women were analysed as part of the statistical shape model in order to add construct validity and avoid over-fitting. This group included women with no cardiovascular disease or risk factors. Propensity score matching was undertaken in R MatchIt, although 1:1 matching was not possible owing to underrepresentation of women of African ancestry in the non-pregnant cohort.

### MRI

After informed consent was obtained, participants had an MRI on a clinical 1.5T Philips Ingenia scanner using a combined 24-channel posterior and torso (dStream) coil. Maternal comfort in the supine position was achieved by careful positioning of the head and legs on elevated cushions, back padding, and additional cushions as requested. The MRI was performed in two sessions (maternal cardiac, and fetal-placental) each of which lasted around 30 minutes. A break was given in the middle of the session. In order to reduce any bias that could arise from maternal anxiety at the start of the scan (for example tachycardia or hypertension), women with an odd case study ID underwent fetal-placental imaging first, and those with an even ID underwent cardiac imaging first.

An obstetrician or midwife was present for all scans. Maternal temperature was recorded before and after the scan. Continuous oxygen saturations were measured, and blood pressure was taken every 10 minutes.

The protocol is illustrated in Supplementary Figure S1 and details of all individual sequences provided in Supplementary Text and Supplementary Table S1; an example of images acquired is given in Supplementary Figure S2.

Non-pregnant controls from the healthy control group underwent an equivalent cardiac MRI protocol on a 1.5-T Philips Achieva system (Best, the Netherlands) as previously described.^29^

### Analysis

All analyses were undertaken by operators masked to the clinical diagnosis. The placenta was visually inspected on a T2 image and reported in line with our previous work.^30^ Manual outlining was performed on a functional scan mapped to subsequent scans by JH (interclass correlation with AH previously confirmed as 0.92). Monoexponential fitting was performed to obtain T2* maps. Mean placenta T2*, histogram skewness and kurtosis were calculated.

Brain volume (supratentorial tissues excluding brainstem and cerebrospinal fluid) was manually segmented on functional maps by DC (interclass correlation previously confirmed as 0.811). Mean T2* values were obtained by averaging the T2* maps from manually outlined brains.

The cardiac MRIs were analysed using cvi42 post-processing software (Version 5.1.4, Circle Cardiovascular Imaging Inc., Calgary, Canada), using standard clinical methodology.^31^

The 3D left ventricular morphology was studied by the construction of a statistical shape model from the segmentations of the short axis stack at the end diastolic frame as reported in previous studies.^32–34^ Briefly, 3D meshes were built using a computational anatomy tool kit,^35^ anatomical modes of variation were found by Principal Component Analysis, and the impact of pre-eclampsia was assessed by an optimised linear discriminant analysis of the first 12 modes.

Flow data was processed using CVI42 Flow version 5.10.3. The magnitude image with the sharpest contrast was used to determine vessel contours. Contours were then propagated to phase contrast images (with manual correction as required) in all temporal phases.

All parameters were first assessed against gestational age, and then as cases against controls. Matching was undertaken to nearest gestational age, with further analysis using the Mann-Whitney U Test.

### Statistical Analysis

Statistical analysis was performed with R version 3.6.0 (R Foundation for Statistical Computing) and RStudio Server version 1.043. Normality was confirmed on histograms. Variables are expressed as percentages if categorical, mean ± SD if continuous and normal, and median (interquartile range) if continuous and non-normal. Baseline anthropometric data were compared by using Kruskal-Wallis tests and, if differences were identified, a Wilcoxon test was used for pairwise comparisons with Benjamini-Hochberg adjustment for multiple testing. Imaging parameters in 2 or more groups were compared by using analysis of covariance, adjusted for relevant clinical covariates including gestational age at time of scan. When differences were significant, a Tukey post hoc test was applied for pairwise multiple comparisons.

## Results

### Study participants

Seventy-eight scans were obtained from 65 pregnant women (with nine women having two scans, and two having three scans). An additional 38 scans were obtained from healthy non-pregnant controls (demographic characteristics provided in Supplementary Table S2). Cardiac assessment was not completed in two pregnant participants: one owing to claustrophobia, and one to worsening hypertension. For a further 16 women, no cardiac data were obtained. Nine women developed pregnancy complications after being scanned as a control and so were excluded. Thirteen women with pre-eclampsia were recruited, one of whom had three MRIs. Of the 13, six had pre-eclampsia superimposed on chronic hypertension. Table 1 summarises the demographics of all cohorts. Supplementary Table S3 details relevant obstetric history and outcomes of the pre-eclamptic cohort.

### Placenta

In the cross-sectional control group, there was a decrease in mean T2* values across gestation (p<0.01). The decrease in mean T2* in the chronic hypertensive group did not reach significance. The pre-eclamptic cohort had significantly lower T2* values throughout gestation, and a gradual decline was not seen. Z-scores for all three groups were calculated for both mean placental T2* and placental volume (Figure 3A-B). For mean T2*, the z-scores obtained were controls = 0.00+-1.03; chronic hypertension -0.71+- 0.7 (p=0.2); pre-eclampsia -1.88+- 0.75 (p<0.001); for the placental volume for controls 0.00+-1.03; for chronic hypertension -0.31 +- 1.025 (p=0.4); for the pre-eclampsia –0.54+-1.08 (p=0.05).

**Figure 1:**
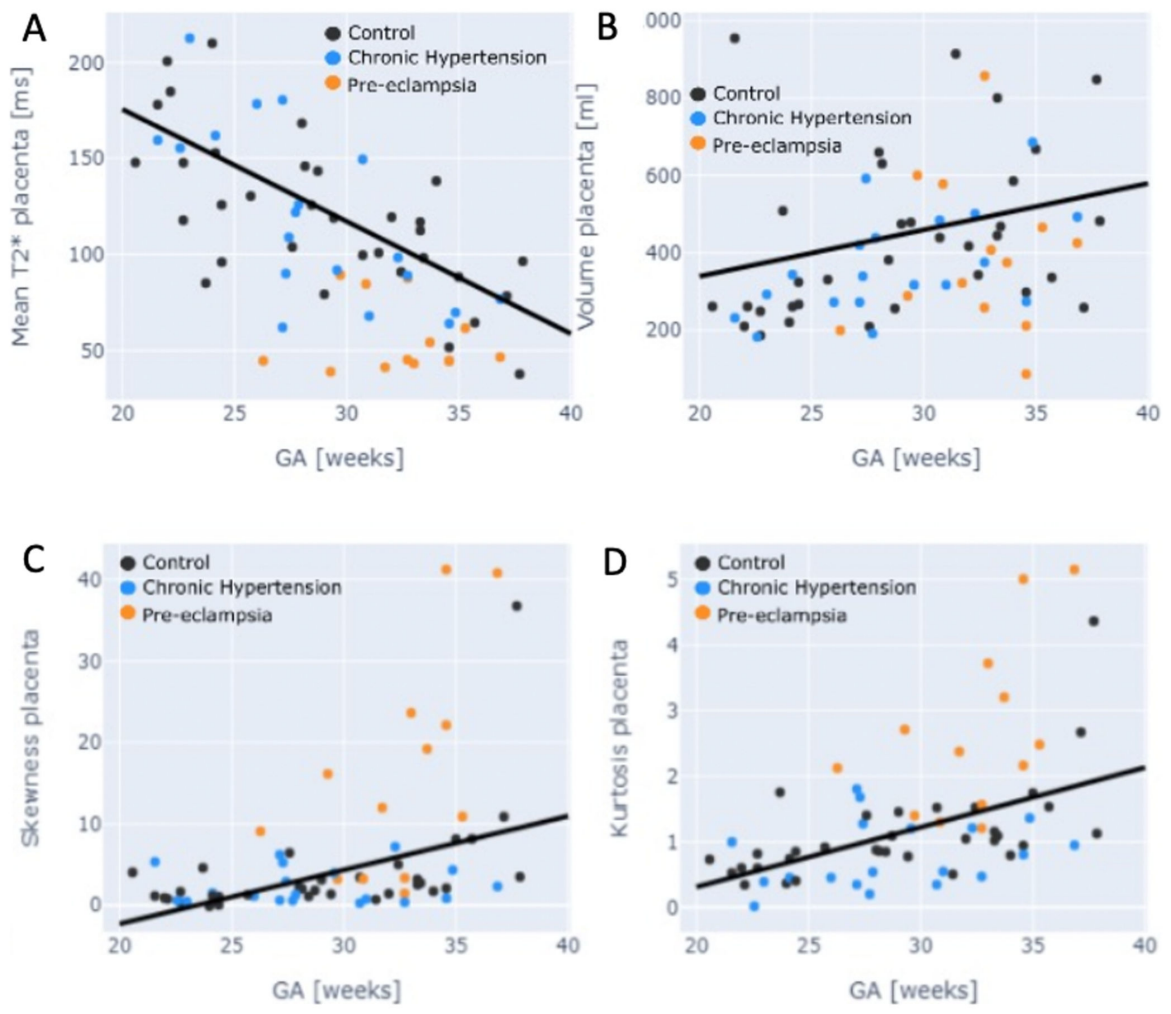
Placental anatomical and T2* by group A) mean placental T2* by gestation; B) placental volumeby gestation; C) skewness and D) kurtosis by gestation. Black dot = control; blue = chronic hypertension; orange = pre-eclampsia.

Both skewness and kurtosis increased throughout gestation in the control group, with the chronic hypertensive group overlying this, although not reaching significance. The pre-eclamptic group had higher skewness and kurtosis than both other groups, and placental volume was reduced (p=0.02) (Figure 1). The Z-scores for skewness for the chronic hypertension group was –1.46+-3.06 and for pre-eclampsia 1.64+-1.75 for kurtosis in the chronic hypertension cohort –0.26-0.77 and for pre-eclampsia 1.67+-1.45. (Figure 3 C-D) 23% with pre-eclampsia had placental T2* values with a z-score of -1 or greater; of note these were the only women without any pre-eclampsia related abnormalities on placental histopathology (Table 2, Supplementary Table S3). These findings were unchanged by removal of longitudinal datasets from the analysis.

Visual inspection of control versus pre-eclamptic placentas revealed a more variable lobule size and less consistent signal intensity in women with pre-eclampsia. There was also an increase in low signal areas as compared to controls.

### Fetal brain

**Figure 2:**
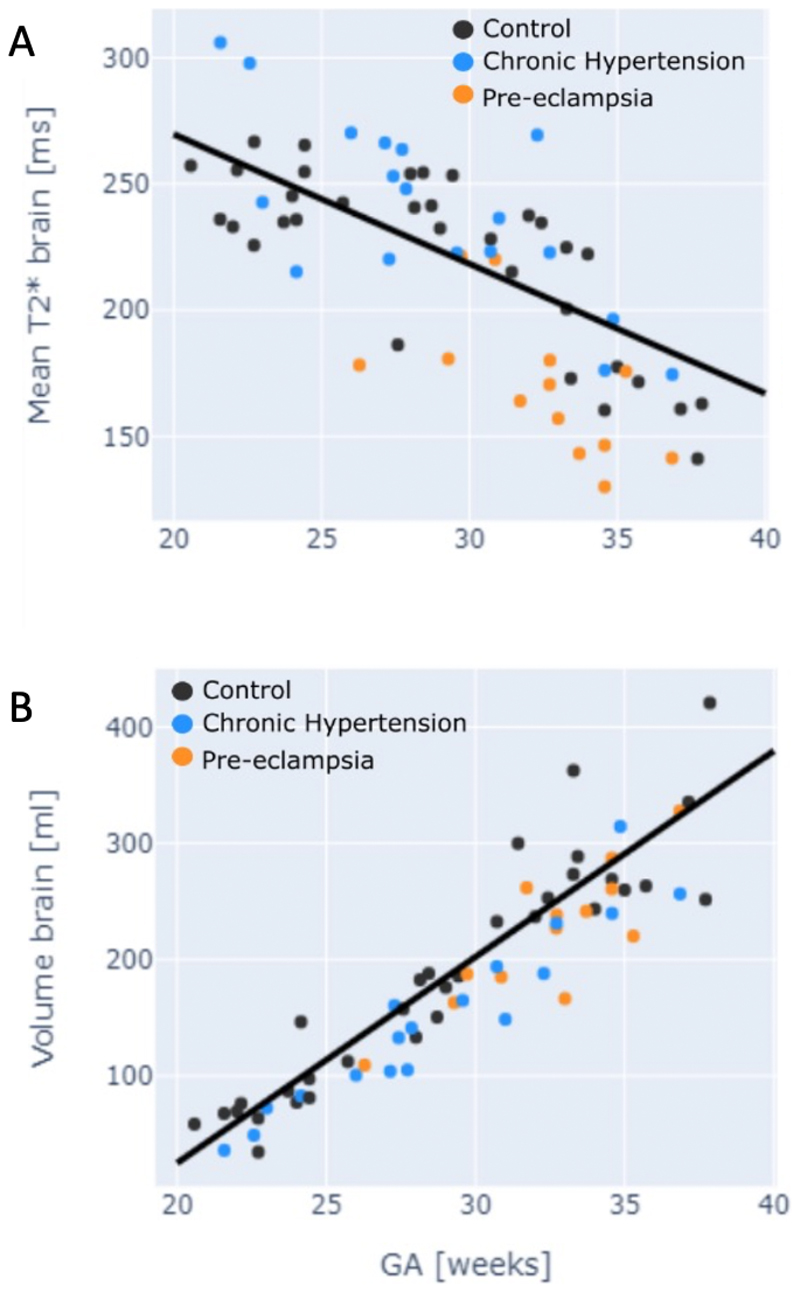
Fetal brain anatomical and T2* by group A) Fetal brain mean T2* by gestation; B) fetal brain volume by gestation. Black dot = control; blue = chronic hypertension; orange = pre-eclampsia.

**Figure 3:**
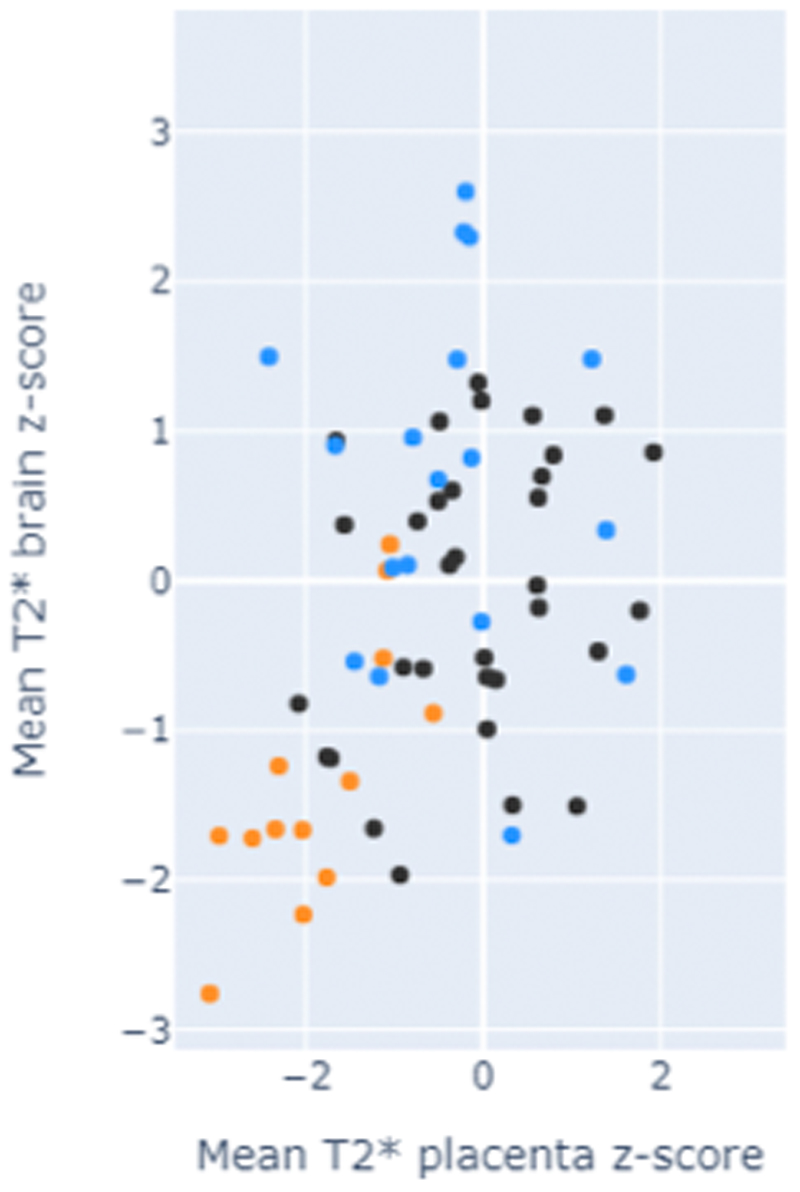
Z-scores for the A) placental mean T2*, B) brain mean T2* are given together with a C) comparison of fetal brain mean T2* z-score and placental mean T2* z-score. Z-scores are given for D) placental volume, E) placental T2* skewness and F) placental T2* kurtosis. Black dot = control; blue = chronic hypertension; orange = pre-eclampsia.

Fetal brain T2* values were demonstrated to decline with advancing gestation among controls. This relationship was preserved in the chronic hypertensive group. Among women with pre-eclampsia, the fetal brain T2* values were significantly reduced through all gestations (p<0.01) and did not have a linear decline with gestation (Figure 2). Z-scores for all three groups were as follows: controls = 0.0+-0.91; chronic hypertension = -0.65+-0.94 (p=0.4); pre-eclampsia = -1.34+-0.84 (p<0.001, Figure 3E) Among pre-eclamptic participants, 31% of cases had brain T2* was in line with controls; in 75% these cases fetal Doppler studies were normal (Table 2, Supplementary Table S3). One case demonstrated an abnormal T2* values but with preserved fetal Doppler studies (Table 2, Supplementary Table S3). Brain volume was reduced in the presence of pre-eclampsia (p=0.03) (Figure 2). These findings were unchanged by removal of longitudinal datasets from the analysis.

Brain and placental T2* z-scores showed a direct correlation between normal and low scores in both domains (Figure 3F).

Major differences in the placental and fetal findings of the control and pre-eclampsia groups are summarised in Supplementary Figure 3.

### Cardiac MR

Systolic (p<0.001) and diastolic (p=0.03) blood pressure were lower in the pregnant controls (107 / 66 mmHg) than in pre-eclamptic participants (121 / 77 mmHg). There were no other baseline anthropometric differences between groups.

**Figure 4:**
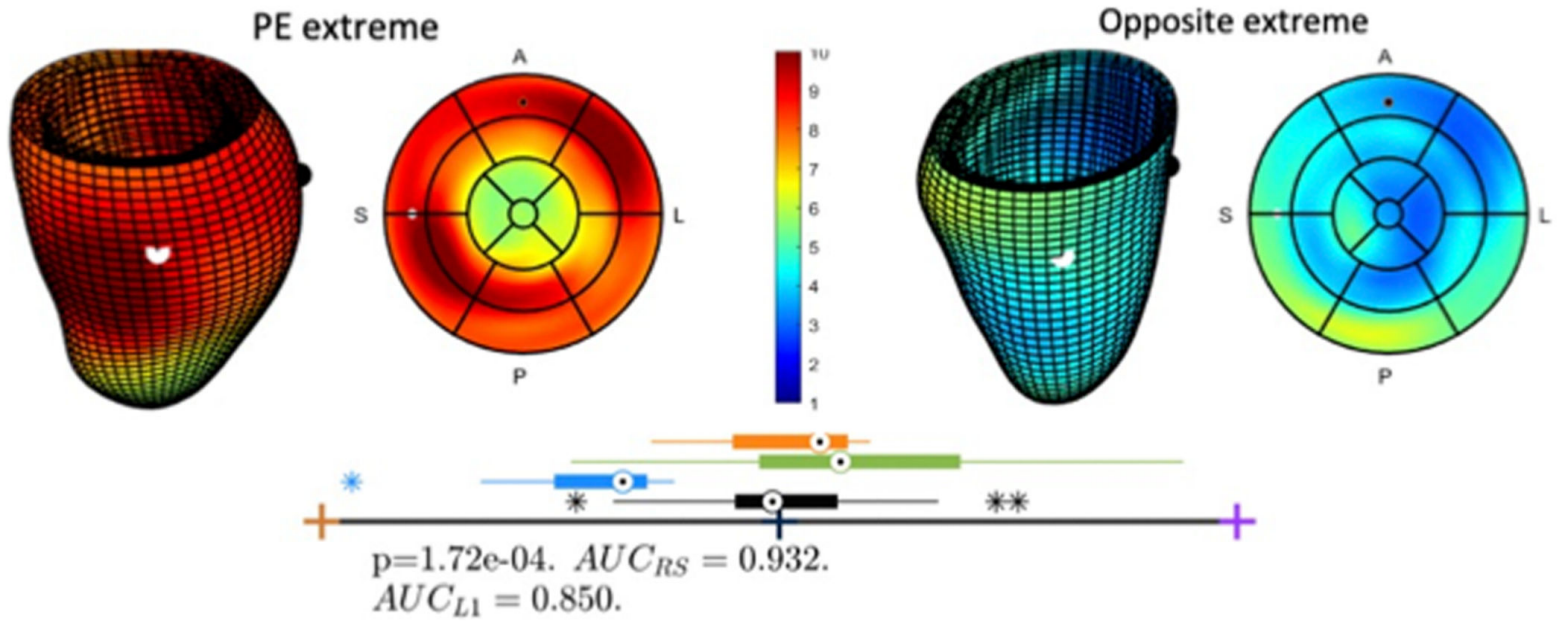
Left ventricular remodelling associated with pre-eclampsia Left ventricular remodelling associated with pre-eclampsia (PE), illustrating thickened walls and eccentricity & bulging located at the outflow track. **Top panel:** extremes of the anatomical mode that best discriminate pregnant controls to pregnant pre-eclamptic subjects - The 3D and bullseye views are colour-coded by thickness (in mm) and have black and white spheres at the sides of the outflow track for easy mapping between both views – A: Anterior; P: Posterior; L: Lateral; S: Septal. **Bottom panel:** box-plot of distributions of pregnant controls (black, n=26), pre-eclampsia (blue, n=9), chronic hypertension (orange, n=4) and non-pregnant controls (green), together with metrics of the discriminant performance of the linear discriminant analysis between pregnant controls and pre-eclampsia (AUC: Area Under the Curve; RS: Resubstitution; L1: Leave-1 out cross-validation).

The LV mass of the pre-eclamptic group (98.05 ± 24.5 g) was higher than both the pregnant controls (83.3 ± 12.08 g; p = 0.04) but not different from those with chronic hypertension (89.3 ± 18.8; p=0.54)). The study of the 3D anatomy revealed a specific thickening pattern (existence of regional increase in wall thickness wall locations of mid antero-lateral and postero-septal) together with changes in eccentricity (the ventricular cross-section displayed a dilation in the axis oriented in the direction of the outflow tract) and the onset of a bulge below the outflow track, see Figure 4. A detailed inspection of the modes of anatomical variation further reveals that the thickening pattern associated to pre-eclampsia was linked to a localised basal concentric remodelling and not to the complementary basal eccentric remodelling (see modes 11 and 13 in Supplementary Figure S4).

## Discussion

### Summary of main findings

We have demonstrated a comprehensive anatomical and functional MRI protocol for cardiac and fetal-placental imaging that is safe in the second and third trimester of pregnancy and acceptable to women, with 97% of scans completed. As far as we are aware, this is the largest set of functional placental MRI data in pre-eclamptic pregnancies, and confirms previous findings suggestive of altered oxygenation and microstructure. We have demonstrated altered T2* in the fetal brain, in proportion to that seen in the placenta. Finally, we have characterised the remodelling pattern of the maternal left ventricle associated with the presence of pre-eclampsia. Conventional cardiac MRI analysis demonstrated higher LVM in pre-eclampsia than in uncomplicated pregnancies, while 3D statistical modelling revealed specific characteristics of eccentricity and outflow track remodelling beyond the thickening of the walls.

### Comparison to other work

The placental phenotype in control pregnancies is in line with previous work: the reduction in T2* across gestation in normal pregnancies has been demonstrated using both our imaging protocols^36^ and those that of others.^37,37^ As previously demonstrated, there is overlap between chronic hypertensive pregnancies and normal pregnancies in terms of mean T2*, skeweness and kurtosis.^20^ Pregnancies affected by pre-eclampsia are typically outliers in all domains. This is to be expected given the higher rate of hypoxic vascular anomalies seen in the pre-eclamptic placentae compared to that affected by chronic hypertension alone.^39^

In all cases where the placental T2* in pre-eclamptic pregnancies lay within the normal range, there was normal placental histopathology and delivery at later gestations, suggesting a less severe clinical phenotype. Pre-existing work on placental perfusion MRI in women with early and late pre-eclampsia, has demonstrated preserved placental perfusion in women with later onset disease.^40^ The decreased placental perfusion seen in the early pre-eclampsia group, as well as histopathological evidence for greater maternal malperfusion in early onset pre-eclampsia as compared to late,^41^ provide basis for the variation seen in our cohort. Whether this is due only to differences in the placenta, or if the cause of later onset pre-eclampsia is perhaps more driven by maternal cardiovascular changes rather than placental pathology remains uncertain.^7^ Nonetheless, our findings go further to support the ability of functional placental MRI to differentiate between disease severities.

Novel to this study is the association between placental T2* and fetal brain T2*. The negative correlation between decreasing brain T2* and increasing gestation in the normal pregnancy is already established, and thought to be secondary to a number of structural changes including decreasing water density and increasing myelination.^24^ Preservation of brain volume with a decrease in T2* in the pre-eclampsia group may reflect a reduction in oxygenation, with or without reduction in angiogenesis. This seems particularly likely given that this does not seem to occur in the absence of reduced placental T2*, and that in all but one case there is corresponding Doppler evidence of cerebral redistribution. Long-term neurodevelopmental and behavioural outcomes of children born to mothers with pre-eclampsia are recognised, and a small study has shown differences in neuroanatomy persisting at 7-10yrs;^42^ although this is difficult to disentangle from the effects of prematurity, our findings may suggest an antenatal antecedent.

Looking into the mother’s health, novel to this study is the 3D thickening pattern of the left ventricle that is found discriminant of the presence of pre-eclampsia. The extra workload in pre-eclampsia is indeed increasing the cardiac mass and mass to volume ratio, as previously reported in much larger echocardiography studies.^6^ This demonstrates the higher power of cardiac MRI to identify differences between small groups, and its role in cardiovascular research in pregnancy. Our preliminary data further reveal an intriguing pattern of thickening, eccentricity and bulging as illustrated in Figure 6. The fact that the chronic hypertension group did not show differences with control pregnancy along this axis suggests that this is an acute maladaptive remodelling process specific to pre-eclampsia. In relationship with this finding, the chronic remodelling of the left ventricle 5 to 10 years after a hypertensive pregnancy manifest as a global concentric (and not eccentric) thickening of the left ventricle.^43^ Intriguingly in the response of pre-eclampsia during pregnancy it is only the base of the heart where this concentric (and not eccentric) thickening pattern is seen (see Supplementary Figure S1). This collection of observations leads us to hypothesize that it is the base of the heart the region that is first recruited and most intensively working in response to pre-eclampsia, and that the rapid onset of hypertension causes a level of uneven thickening pattern and septal bulging. Given that this study includes both early and later onset cases of pre-eclampsia the similarity in cardiac output between pre-eclampsia and control groups is to be expected.

### Strengths and limitations

This study is the largest to date using MRI to investigate maternal, fetal and placental impactions of pre-eclampsia, using an optimised protocol that is safe and acceptable to women. The data is made more robust by consistent positioning and imaging protocols, as well as post-processing including motion correction. Inclusion of women with chronic hypertension demonstrates where phenotypes are driven by pre-eclampsia specifically.

Limitations include the relatively small number of wome with pre-eclampsia included, reflecting the difficulty recruiting given the often short interval from diagnosis to delivery; this may obscure significant differences between groups, but also limits any comments that can be made on confounding factors in cases such as diabetes and timing of disease onset. Given the small numbers of women with pre-eclampsia included and the variation in phenotype displayed, a multi-dimensional analysis of the heart, placenta and fetal brain is not plausible at this stage.

### Implications

While clinical fetal MRI is largely confined to assessment of structural anomalies, there is evidence here of a potential role for other high risk obstetric conditions. We have demonstrated feasibility of a comprehensive fetal, placental and maternal cardiac MRI in terms of safety, data acquisition and acceptability to women.

Future work into determining risk of developing, likely clinical severity and implications of pre-eclampsia should combine MRI, obstetric ultrasound and biomarkers such as placental growth factor, as it is likely that a combination of investigations could yield greatest information regarding initial diagnosis as well as predicted course of disease.

While we have demonstrated a correlation of findings associated with pre-eclampsia, further work should be done in defining this phenotype. As well as increasing the cohort size, attention should be paid to early and late onset disease, as these are likely to be mechanistically different and have different clinical end point.^44^ Furthermore, an increase in cohort size would allow for separate analysis of previously normotensive women who develop pre-eclampsia, and women with pre-existing chronic hypertension who develop superimposed pre-eclampsia. While fetal growth restriction has previously been associated with reduced placental T2*,^23,44^ we have been unable to delineate the implications of pre-eclampsia on this finding. Creation of a cohort where the interplay between these three findings could be investigated would be of value. MRI could have implications for earlier diagnosis of pre-eclampsia; high-risk women (for example those with early onset growth restriction) who are not yet diagnosed with pre-eclampsia should be included in future work, as this could have significant implications for their management. Moreover, the 3D specific remodelling pattern linked to pre-eclampsia could have implications for prognosis and risk models for future cardiovascular events. Any future work on cardiac imaging should include longitudinal and postnatal data in order to better delineate the mechanisms of continued cardiac risk, and for stratification of risk in individuals.

In terms of perinatal outcomes, we have demonstrated a relationship between pre-eclampsia and reduced fetal brain T2*, and also between reduced fetal brain T2* and fetal cerebral Doppler redistribution. While there is some evidence of abnormal neurocognitive outcomes in children who have been affected by redistribution^46^, further neurocognitive follow up of children with paired MRI and ultrasound data could improve understanding of the longer-term clinical implications of pre-eclampsia on the child.

In terms of techniques used, while the relationship between T2* and deoxyhaemoglobin is well established, it is subject to influence from other factors. Therefore, additional functional imaging, such as diffusion techniques, linked with recent advanced analysis techniques^47^ may lead to greater insight of the mechanisms of pre-eclampsia.

## Perspectives

We demonstrate that MRI may provide maternal and fetal insights into pre-eclampsia that cannot be obtained by other means during pregnancy; and that these can be obtained in a clinical MRI scanner in an examination that is acceptable to women. Functional placental MRI may be able to differentiate between pre-eclampsia disease severities, while fetal brain imaging may help refine neurodevelopmental prognostic assessment of children exposed to pre-eclampsia in utero, as well as give insight into pathological pathways underlying the changes seen in this group. In our small sample, specific 3D pattern of left ventricular remodelling and thickening was found to be discriminant of the presence of pre-eclampsia and may provide a basis for improved clinical risk stratification. Confirmation of these findings in a larger group of women is required prior to inclusion in risk stratification models.

## Supplementary Material

Figure S1

Figure S2

Figure S3

Figure S4

Graphical Abstract

Supplemental Material

## Figures and Tables

**Table 1:** Demographics of participants

Cohort	Pregnant controls (n=29)	Pre-eclampsia (n=13)	Chronic hypertension (n=18)	Retrospectively excluded (n=9)
**Age (years),** **median (IQR)**	34.4 (31.8-37.3)	33.5 (30.8-37.8)	36.8 (33.8-41.5)	33.1 (31.9-35.3)
**Parity**				
**Nulliparous (%)**	13 (45)	11 (85)	5 (28)	6 (67)
**Multiparous (%)**	16 (55)	2 (15)	13 (72)	3 (33)
**BMI (kg/m^2^), **	24.7	28.1	31.9	23.1
**median (IQR)**	(22.9-27.2)	(24.1-33.2)	(27.4-33.8)	(21.9-24.4)
**Smoker**				
**Previous pre-eclampsia (%)**	0	3 (23)	2(8)	0
**Mode of conception**				
**Spontaneous (%)**	26 (90)	12 (92)	4 (22)	9 (100)
**Assisted (%)**	3 (10)	1 (8)	14 (88)	0
**Gestational age at the time of scan (weeks), median (IQR)**	28.1 (23-5-32.1)	32.7 (30.0-34.1)	27.8 (25.0-32.0)	27.3 (24.4-33.3)
**Gestational age at birth, mean (SD)**	39.6 (1.07)	33.4 (2.77)	37.8 (0.7)	36.3 (2.87)
**Mode of delivery**				
**Vaginal**	18 (62)	1 (8)	4 (22)	4 (44)
**Prelabour **				
**Caesarean Section**	4 (17)	11 (84)	8 (44)	3 (33)
**Caesarean Section in labour**	7 (24)	1 (8)	6 (33)	2 (22)
**Birth weight (grams), mean (SD)**	3394 (402)	1781 (552)	2973 (636)	2370 (978)
**Birth weight (grams), range**	2710-4175	695-3070	1560-4840	1010-3220

BMI: body mass index; IQR: interquartile range

**Table 2:** Relationship between fetal brain and placental T2* and clinical parameters in patients with pre-eclampsia *Where placental histopathology is referred to as abnormal this is in reference to pre-eclampsia related pathological findings of the Amsterdam criteria* (maternal vascular malperfusion lesions of the placental bed, including infarcts, retroplacental haemorrhage, distal villous hypoplasia, accelerated villous maturation, and decidual arteriopathy). *Middle cerebral artery pulsatility index below the 5th centile or a cerebroplacental ratio below the 5th centile were used to define cerebral redistribution; while the middle cerebral artery was used clinically to define redistribution in these patients, the decision to include the CPR is reflective of its higher specificity in detecting fetal hypoxia*. Low placental and brain T2* is defined as z-score <-1.

Participant ID	Placental T2*	Placental histopathology	Fetal brain T2*	Fetal Dopplers	Fetal growth restriction
**1**	Low	Abnormal	Low	Redistribution	Yes
**2**	Low	Other vascular abnormality	Low	Normal	Yes
**3**	Low	Abnormal	Low	Redistribution	Yes
**4**	Low	Abnormal	Normal	Redistribution	No
**5**	Normal	Normal	Normal	Normal	No
**6**	Low	Abnormal	Low	Redistribution	Yes
**7**	Low	Abnormal	Low	Redistribution	Yes
**8**	Normal	No pre-eclampsia related changes	Normal	Normal	Yes
**9**	Low	Not available	Low	Redistribution	No
**10**	Low	Abnormal	Low	Redistribution	Yes
**11**	Low	Abnormal	Low	Redistribution	Yes
**12**	Low	Abnormal	Low	Redistribution	Yes
**13**	Normal	Normal	Normal	Normal	No

## Novelty and relevance

### What is new?

- We propose a comprehensive MRI protocol for maternal cardiac and fetal-placental imaging that is safe in pregnancy and acceptable to women, and report on the largest set of functional placental MRI data in pre-eclamptic pregnancies.

### What is relevant?

- The findings suggest altered oxygenation and microstructure in the placenta, which is associated with similar changes in the fetal brain, and identifies a specific pattern of remodelling in the maternal left ventricle that is associated with pre-eclampsia.

### Clinicopathological correlation

- As well as giving insight into the immediate pathophysiological impact of pre-eclampsia, MRI holds potential as a valuable tool for risk stratification both antenatally, and when considering longer-term cardiovascular risk in women.

## Non-standard abbreviations and acronyms

bpm: beats per minute
CO: cardiac output
EDV: end diastolic volume
ESV: end systolic volume
FGR: fetal growth restriction
FOV: field of view
GCS: global circumferential strain
GLS: global longitudinal strain
GRS: global radial strain
IQR: interquartile range
LV: left ventricular
LVM: left ventricular mass
TE: echo time
TR: repetition time
