# Supplementary figures and images for: Pre-eclampsia associated differences in the placenta, fetal brain and maternal heart can be demonstrated antenatally: An observational cohort study using MRI
Source: Hypertension. Author manuscript; Available in PMC 2024 Apr 1. (PMC7615760; doi:10.1161/HYPERTENSIONAHA.123.22442)

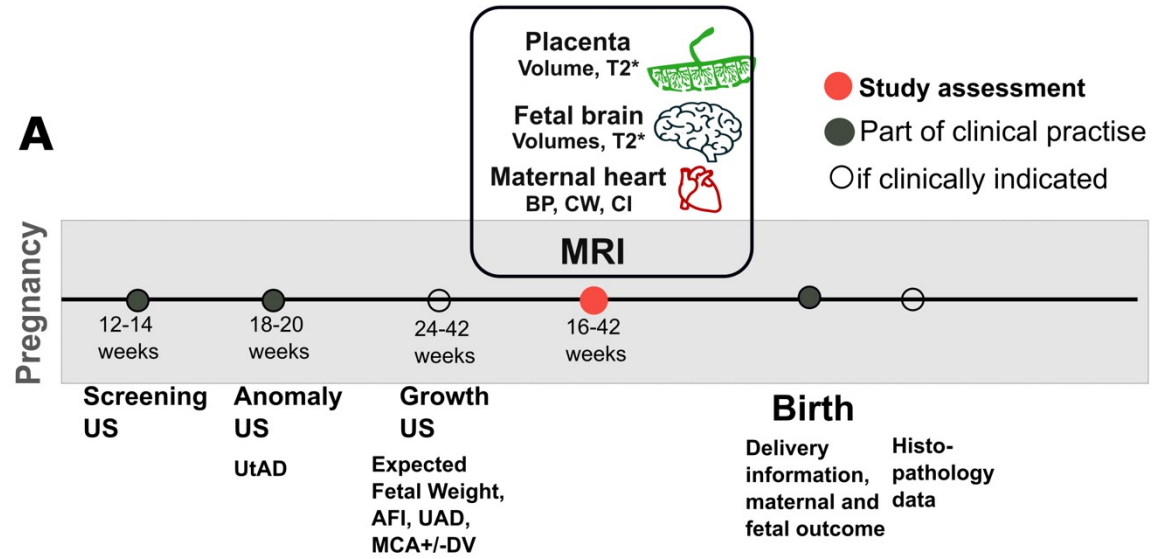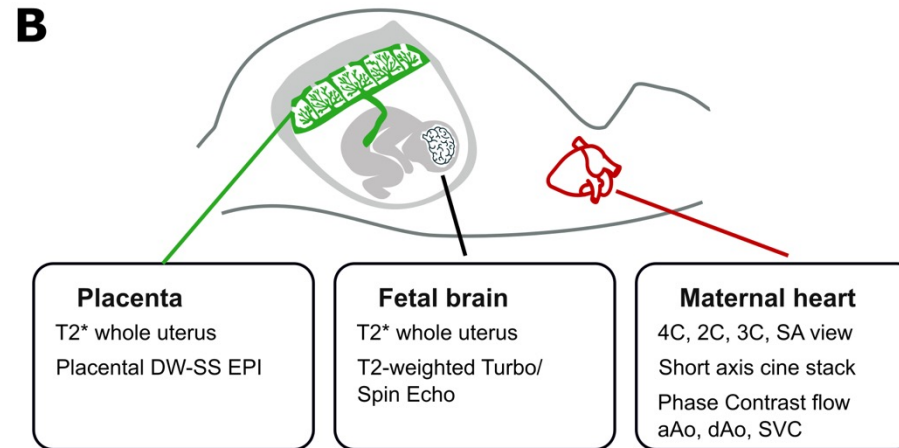

Supplement: Figure S1 [file EMS193471-supplement-Figure_S1.pdf]

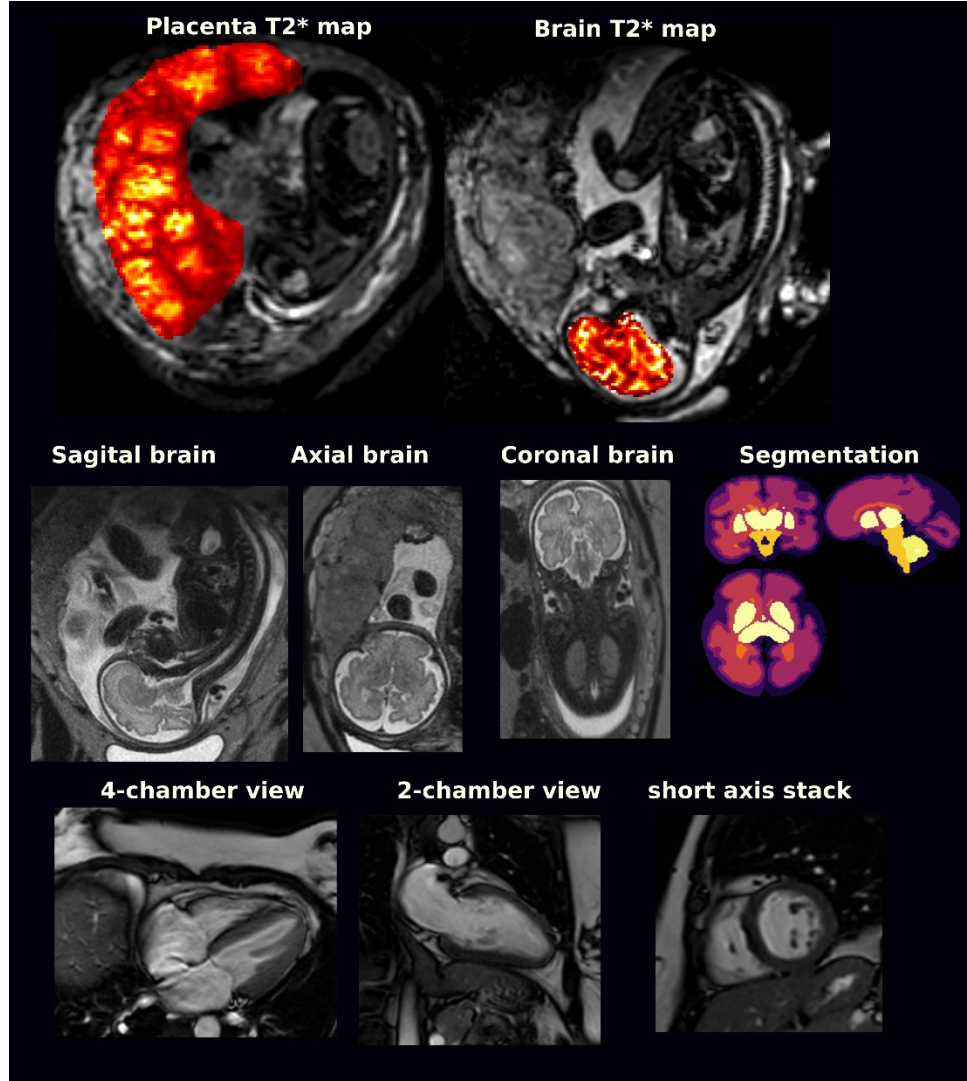

Supplement: Figure S2 [file EMS193471-supplement-Figure_S2.pdf]

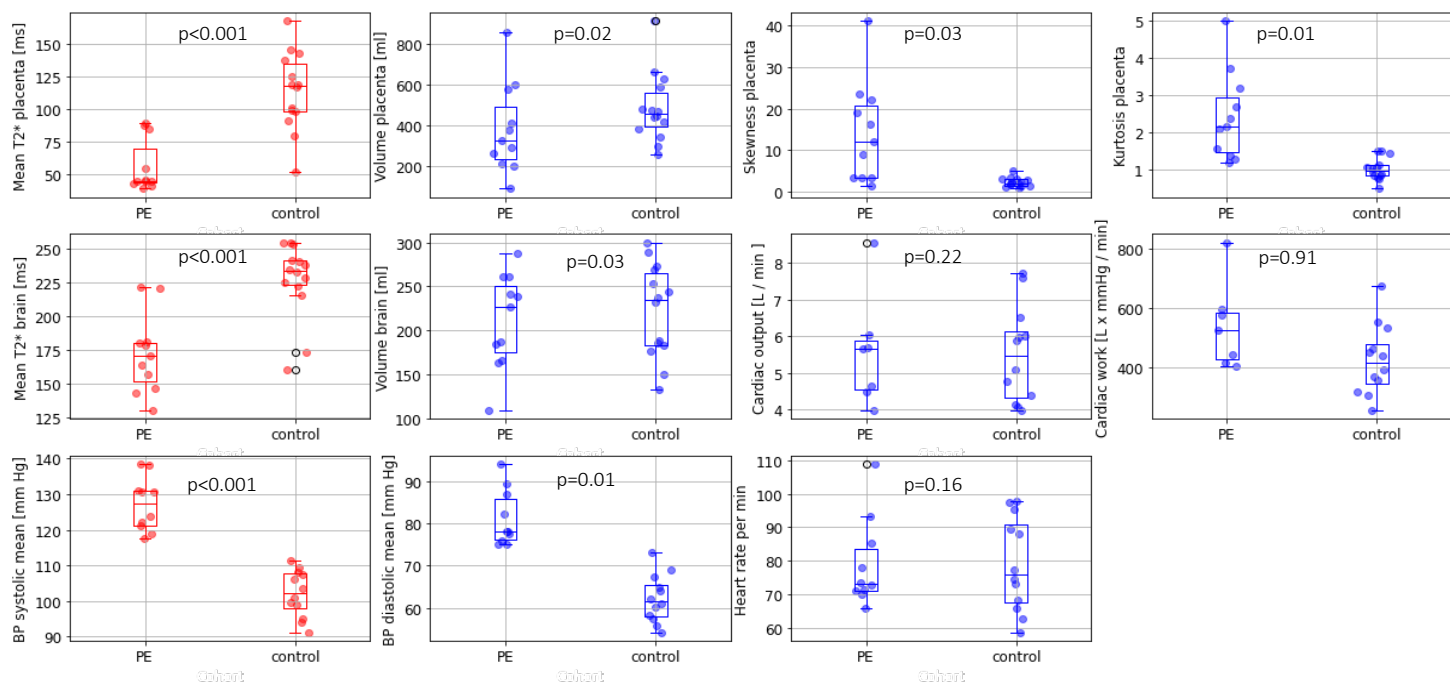

Supplement: Figure S3 [file EMS193471-supplement-Figure_S3.pdf]

Mode -3std

11

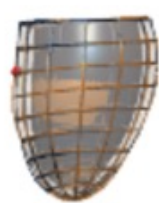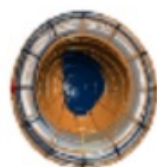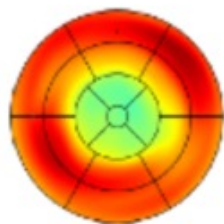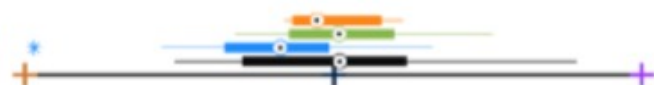

+3std

13

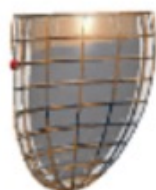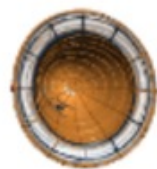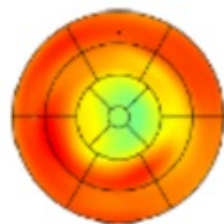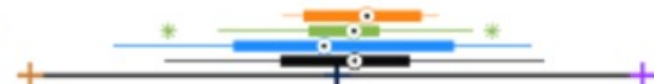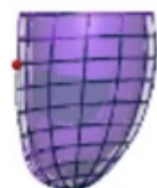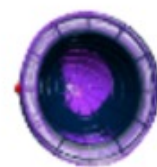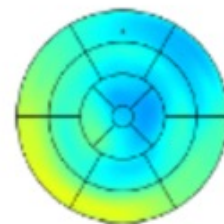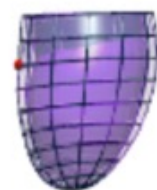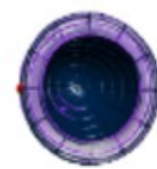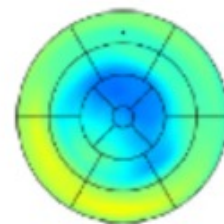

Supplement: Figure S4 [file EMS193471-supplement-Figure_S4.pdf]

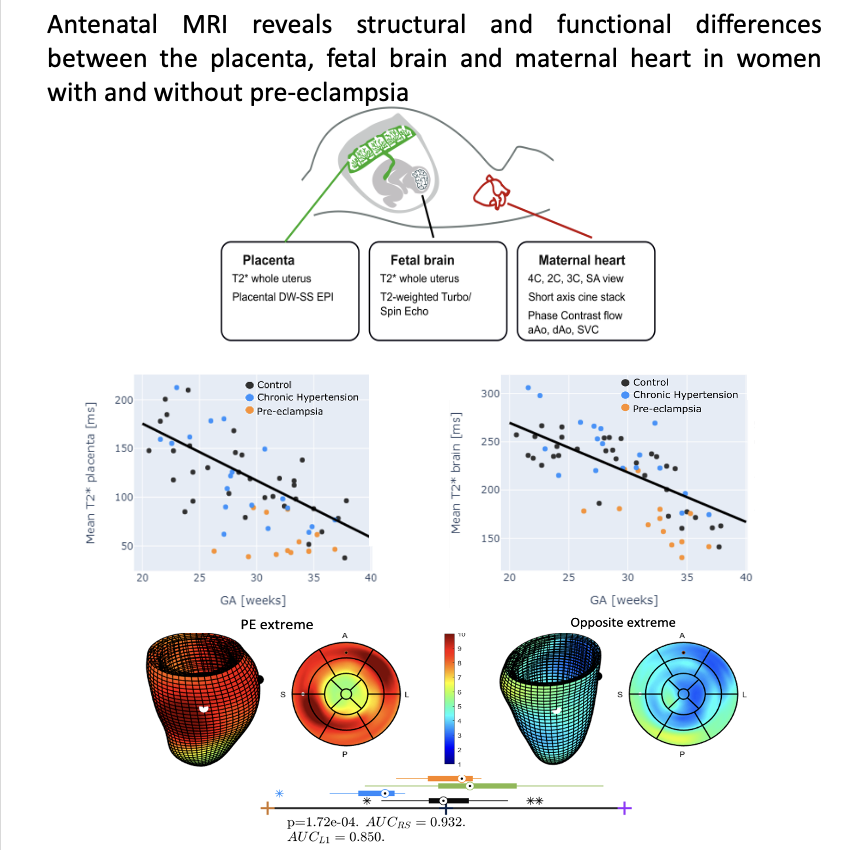

Supplement: Graphical Abstract [file EMS193471-supplement-Graphical_Abstract.png]
