# Supplementary material for: Pre-eclampsia associated differences in the placenta, fetal brain and maternal heart can be demonstrated antenatally: An observational cohort study using MRI
Source: Hypertension. Author manuscript; Available in PMC 2024 Apr 1. (PMC7615760; doi:10.1161/HYPERTENSIONAHA.123.22442)
Supplement: Supplemental Material [file EMS193471-supplement-Supplemental_Material.docx]

**Supplementary material to:**

**Pre-eclampsia associated differences in the placenta, fetal brain and maternal heart can be demonstrated antenatally: An observational cohort study using MRI.**

**Short title: Maternal-fetal changes in pre-eclampsia**

Megan Hall MRCOG, BSc (Hons)^1,2^, Antonio de Marvao MRCP, PhD^1,3,4^, Ronny Schweitzer MD^3,4^, Daniel Cromb MRCPCH, BSc (Hons)^2,^, Kathleen Colford, BSc (Hons), PGDip^2^, Priya Jandu, MSc^5^, Declan P O'Regan, FRCP, FRCR, PhD^4^, Alison Ho, MRCOG, PhD^1,2^, Anthony Price, PhD^2,6^, Lucy C. Chappell, FRCOG, PhD, FMedSci^1^, Mary A. Rutherford, MRCPCH, FRCR, MD^2^, Lisa Story MRCOG, PhD^1,2^, Pablo Lamata, PhD^6^, Jana Hutter, PhD^2,6^

1. Department of Women and Children’s Health, King’s College London, UK
2. Centre for the Developing Brain, King’s College London, UK
3. School of Cardiovascular Medicine, King’s College London, UK
4. MRC London Institute of Medical Sciences, Imperial College London, UK
5. GKT School of Medical Education, King’s College London, UK
6. Centre for Medical Engineering, King’s College London, UK

Corresponding author: Megan Hall

First Floor, South Wing

St Thomas’ Hospital

London

SE1 7UH

[megan.hall@kcl.ac.uk](mailto:megan.hall@kcl.ac.uk)

+447906542307

**Supplementary Text: Details of acquisition protocols**

*Fetal-placental MRI*
Fetal brain and whole uterus structural imaging was performed in five planes (transverse centred on fetal brain; two opposing sagittal oblique planes; maternal coronal plane and maternal sagittal plane) in order to obtain whole uterus and dedicated placental images. MRI was undertaken using 2D-single-shot Turbo Spin Echo (resolution 1.5x1.5x2.5mm, field of view (FOV) 320x320x110mm, echo time (TE) 180ms). Fetal body imaging was performed for clinical reporting only.

Placental T2* mapping was obtained using a multi-echo gradient-echo sequence (2.5mm isotropic, FOV=300x300x110mm, TE=11, 58, 117, 176ms). Placental diffusion-weighted imaging used a single-shot echo-planar-imaging sequence with parameters adapted to meet the expected diffusivity (1 b=0, 6 b=375, 6 b=750; transverse relaxation (TR)=6.6ms, TE=78ms, matrix = 512x512x56, resolution 2x2x4mm). Both sequences were repeated twice to improve data robustness.

*Cardiac MRI*
Anatomical and functional cardiac MRI sequences were acquired using commonly employed sequences adapted to reduce acoustic noise and heating, and so improve suitability in pregnancy. Views taken included: 4 chamber, 2 chamber, 3 chamber, short axis and outflow (acquired using dynamic balanced steady state free precession sequences with 30 cardiac phases, 11 second breath-hold, FA=60, TE/TR~2/4ms, resolution=1.7x1.7x8mm); and a short axis stack (14 slices, sense=2, resolution 2x2x10mm). Phase contrast flow sequences of the ascending and descending aorta, and the superior vena cava were acquired with individually adapted encoding velocities (30 phases, 14 second breath-holds, sense=2, resolution=2.5x2.5x8mm). A pragmatic decision was made to obtain inferior vena cava images during the ‘fetal-placental’ portion of the MRI as the coil was appropriately placed.

**Supplementary Table S1: Scanning parameters**

| **Placental and Fetal MRI** | | |
| --- | --- | --- |
| **Anatomical** | **T2 weighted Turbo Spin Echo**  TR 30s; TE 180ms; Matrix size 384x384x150; Resolution 1.25x1.25x2.5mm | Structural imaging of the fetus and placenta |
| **T2* static** | **Multi-Echo Gradient Echo**  TR 23s; TE 12.599ms/65.34ms/118.08ms/170.821ms/223.562ms; Dynamics – 2; Matrix size 224x224x850; Resolution 2.5x2.5x2.5mm | Proxy marker of tissue oxygenation |
| **Diffusion** | **Pulsed Gradient Echo Spin Echo, Echo Planar Imaging**  TR 6.6s; TE 78ms; Matrix 512x512x56; Resolution 2x2x4mm; b 375 (6 directions); b 750 (6 directions) | Exploration of tissue microstructure |
| **T2* dynamic** | **Multi-Echo Gradient Echo**  TR 6.4s; TE 7.84ms/60.574ms/113.308ms/116.041ms; Dynamics – 30; Matrix size 256x256360; Resolution 2.5x2.5x2.5mm | Proxy marker of tissue oxygenation |
| **Cardiac MRI** | |  |
| **Cine bSSFP** | **bSSFP**  30 phases; FA 60; TR 4ms; TE 2ms; Resolution 1.7.1.7.8mm; ~11 second breath-hold | Cardiac anatomy and strain |
| **Short axis stack** | **bSSFP**  TR 4.1s; TE 2ms; 14 slices; Sense 2; Resolution 2x2x10mm | Cardiac anatomy and strain in view complementary to that above |
| **Phase contrast (breath-hold option)** | 30 phases; Sense 2; Resolution 2.5x2.5x8mm; 14 second breath-hold | Functional assessment of blood flow |
| **Phase contrast (free breathing option)** | 30 phases; Sense 2; Resolution 2.5x2.5x5mm; NSA 4 | Functional assessment of blood flow |

*TR: repetition time; TE echo time; bSSFP balanced Steady State Free Precession*

**Supplementary Table S2: Demographic characteristics of non-pregnant control cohort**

| **Cohort (number of scans)** | **Non-pregnant controls (n=38)** |
| --- | --- |
| Age (years), median (IQR) | 32.0  (27.0-39.0) |
| BMI (kg/m^2^), median (IQR) | 25.3  (21.2-25.4) |
| Previous pre-eclampsia (%) | 0 |

**Supplementary Table S3: Medical history and obstetric outcomes of the pre-eclamptic cohort**

| **Ethnicity (n)**  White  Black  South Asian  Other | 2  5  4  2 |
| --- | --- |
| **Parity (n)**  Nulliparous  Parous | 9  4 |
| **Gestational age at PET diagnosis (weeks) (range)** | 27.0 (20.7 – 35.0) |
| **Pre-eclampsia superimposed on chronic hypertension (n)** | 6 |
| **PlGF (pg/ml) (n)**  >12  <12 | 2  2 |
| **Proteinuria (n)** | 13 |
| **Acute kidney injury (n)*** | 5 |
| **Hepatic dysfunction (n)*** | 8 |
| **Haemolysis (n)*** | 1 |
| **Neurological involvement (n)*** | 0 |
| **Thrombocytopaenia (n)*** | 2 |
| **Fetal growth restriction (n)** | 9 |
| **Gestational age at MRI (weeks) (range)** | 32.0 (26.3 – 35.3) |
| **Antenatal aspirin use (n)** | 7 |
| **Antihypertensives at time of MRI (n)**  Single agent  Two agents  Three agents | 8  4  1 |
| **Maximum antihypertensives (n)**  Single agent  Two agents  Three agents  Four agents | 5  4  3  1 |
| **Evidence of redistribution on ultrasound at time of MRI (n)**  Yes  No | 9  4 |
| **Mode of delivery (n)**  Caesarean section in labour  Caesarean section prior to labour  Spontaneous vaginal | 2  10  1 |
| **Indication for delivery (n)**  Maternal  Fetal | 4  9 |
| **Neonatal complications (n)**  NICU admission  Respiratory support  Necrotising enterocolitis  Hypoglycaemia  Jaundice | 12  4  2  5  6 |
| **Placental histopathology (n)**  Abnormalities consistent with pre-eclampsia  Other abnormalities  Normal  Not available | 8  2  2  1 |

PlGF: placental growth factor.

*Diagnosed at any stage in pregnancy or postnatal period

**Supplementary Figure S1: Schematic of study protocol**

*US: ultrasound; UtAD: uterine artery Doppler; AFI: amniotic fluid volume; UmbAD: umbilical artery Doppler; MCA: middle cerebral artery Doppler; DV: ductus venosus Doppler; BP blood pressure; CW cardiac work; CI: cardiac index; DW-SS EPI: diffusion weighted single shot echo planar imaging; 4C: 4 chamber; 2C 2 chamber; 3C 3 chamber; SA: short axis; aAo ascending aorta; dAo descending aorta; SVC superior vena cava.*


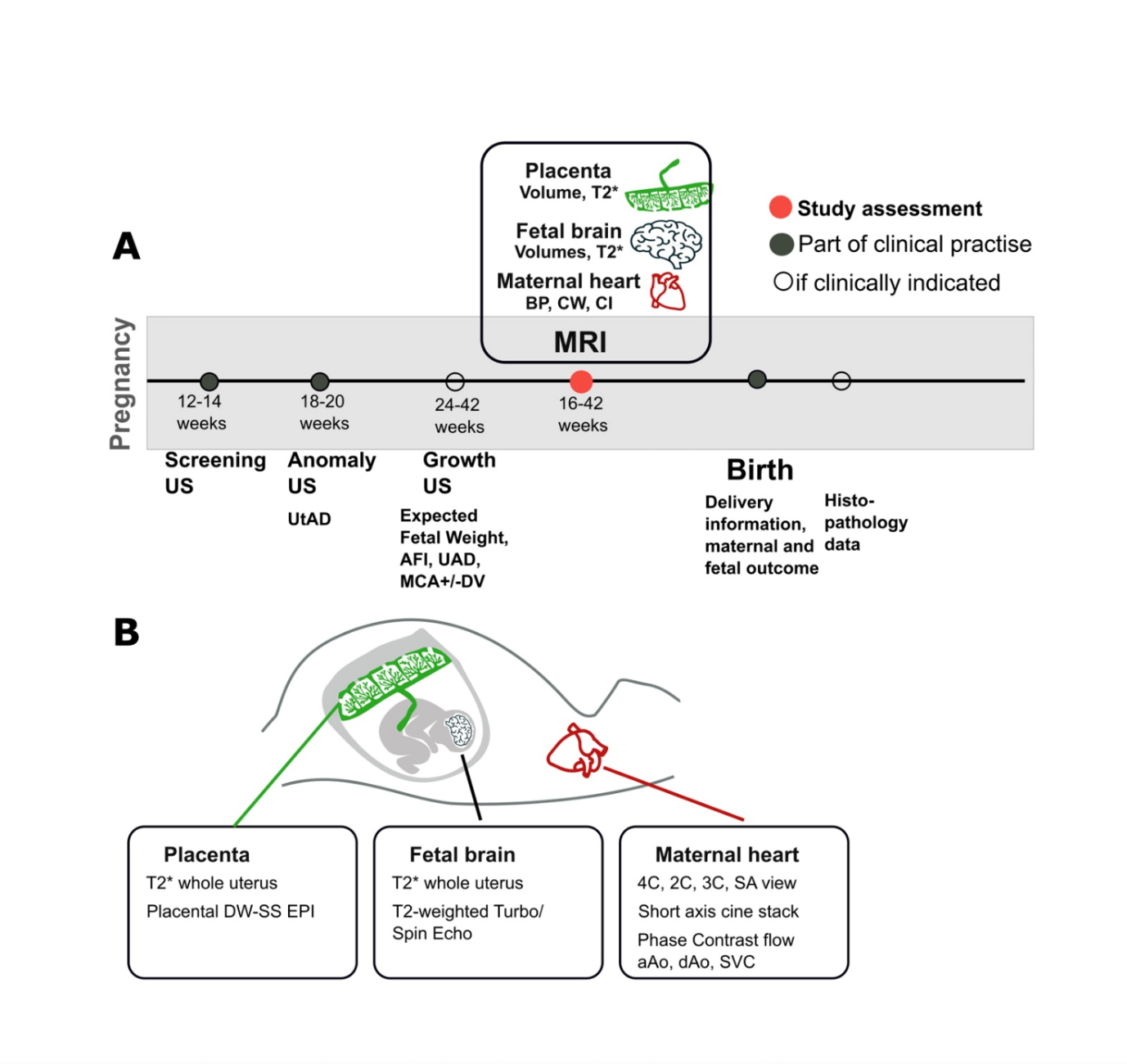


**Supplementary Figure S2: Summary of images acquired on protocol**

*Top panel: placental and fetal brain T2* maps superimposed on a coronal image*

*Middle panel: three views of the fetal brain obtained as part of this study, and the corresponding segmentation obtained after motion correction and manual segmentation*

*Bottom panel: cardiac views obtained.*


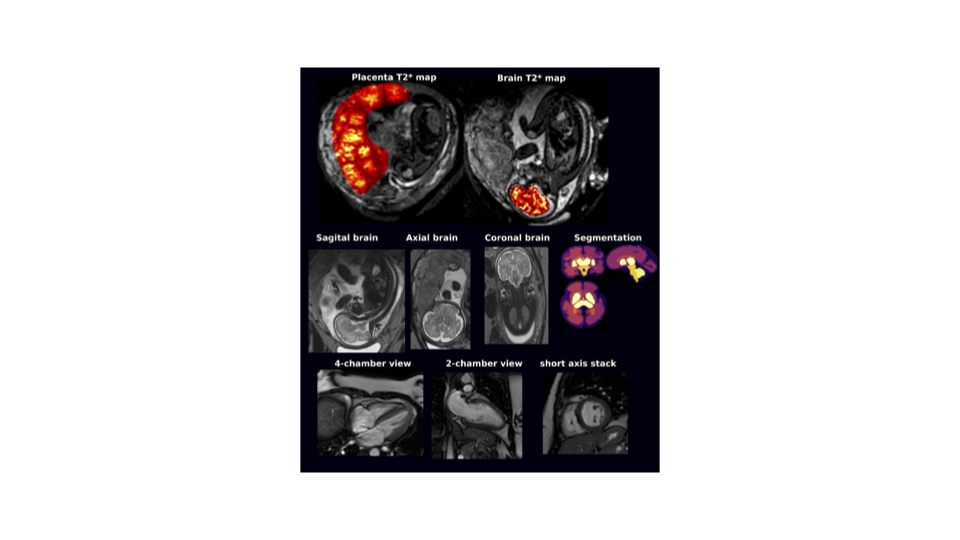


**Supplementary Figure S3: Summary of major findings in the pregnant control (non-hypertensive) and pre-eclamptic cohorts**


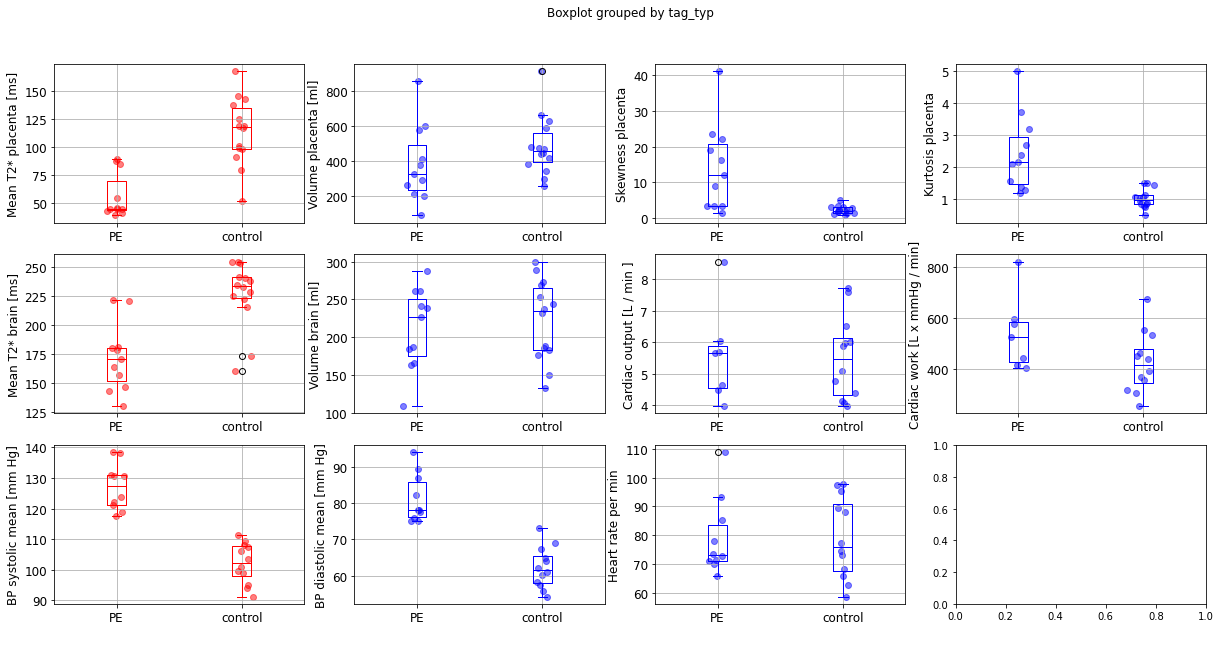


p<0.001

p=0.02

p<0.001

p=0.03

p=0.01

p=0.03

p=0.22

p=0.91

p<0.001

p=0.01

p=0.16

*Placental mean T2* p<0.001; placental volume p=0.02; placental T2* skewness p=0.03; placental T2* kurtosis p=0.01.*

*Fetal brain mean T2* p<0.001; fetal brain volume p=0.03.*

*Cardiac output p=0.22; cardiac work p=0.91; mean systolic blood pressure p<0.001; mean diastolic blood pressure p=0.01; mean heart rate p=0.16*

*BP: blood pressure*

**Supplementary Figure S4: Anatomical models of variation that most correlate with thickness**


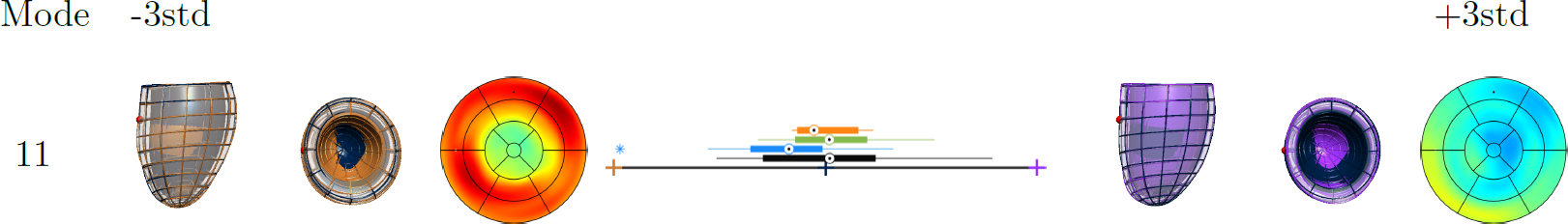


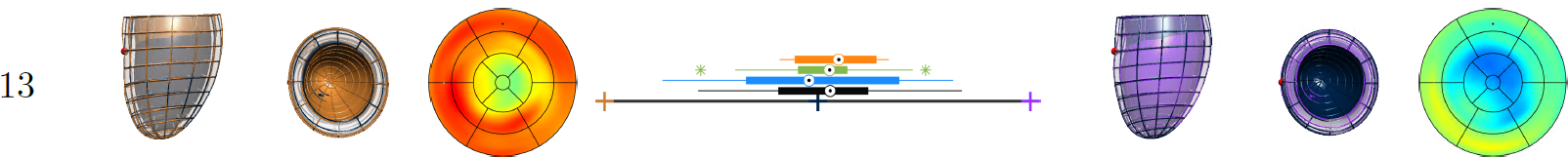


Sup. Fig. 1: The two anatomical modes of variation that most correlated with thickness (R2=22.89% and 26.58% respectively for mode 11 and 13), with mode 11 showing a concentric basal thickening (narrower cavity at the base) and with mode 13 showing a complementary eccentric basal thickening (wider cavity at the base). Box-plots of the subjects (pregnant control: black; pre-eclampsia: blue; non-pregnant control: green; chronic hypertension: orange) show an almost significant difference in mode 11 between pregnant control and pre-eclampsia (p=0.07) but the complete overlap in mode 13 (p=0.77).
